# Supplementary material for: Whole genome sequencing of Rhodotorula mucilaginosa isolated from the chewing stick (Distemonanthus benthamianus): insights into Rhodotorula phylogeny, mitogenome dynamics and carotenoid biosynthesis
Source: PeerJ. 2017 Nov 14;5:e4030. doi: 10.7717/peerj.4030 (PMC5691792; doi:10.7717/peerj.4030)
Supplement: Table S1 [file peerj-05-4030-s001.docx]

Searching for homologs of sequence RIT389_ITS It should take at most 18 seconds. Please wait...

Database(s) used: UNITE (fungi) + INSD ( = GenBank, EMBL, DDBJ) + Envir.

BLASTN 2.2.29+

Reference: Stephen F. Altschul, Thomas L. Madden, Alejandro A. Schaffer, Jinghui Zhang, Zheng Zhang, Webb Miller, and David J. Lipman (1997), "Gapped BLAST and PSI-BLAST: a new generation of protein database search programs", Nucleic Acids Res. 25:3389-3402.

Database: data/unite_full_blastn.fas

763,178 sequences; 435,288,657 total letters

Query= RIT389_ITS

Length=774

Query= RIT389_ITS

Length=774

Score E

Sequences producing significant alignments: (Bits) Value

| [KF411559](https://unite.ut.ee/bl_forw.php?id=396262) | Rhodotorula | mucilaginosa | 1397 | 0.0 |
| --- | --- | --- | --- | --- |
| [KF411558](https://unite.ut.ee/bl_forw.php?id=396263) | Rhodotorula | mucilaginosa | 1397 | 0.0 |
| [KF411556](https://unite.ut.ee/bl_forw.php?id=396265) | Rhodotorula | mucilaginosa | 1397 | 0.0 |
| [KF411555](https://unite.ut.ee/bl_forw.php?id=396266) | Rhodotorula | mucilaginosa | 1397 | 0.0 |
| [KF411554](https://unite.ut.ee/bl_forw.php?id=396267) | Rhodotorula | mucilaginosa | 1397 | 0.0 |
| [KF411553](https://unite.ut.ee/bl_forw.php?id=396268) | Rhodotorula | mucilaginosa | 1397 | 0.0 |
| [KF411552](https://unite.ut.ee/bl_forw.php?id=396269) | Rhodotorula | mucilaginosa | 1397 | 0.0 |
| [KF411551](https://unite.ut.ee/bl_forw.php?id=396270) | Rhodotorula | mucilaginosa | 1397 | 0.0 |
| [KF411550](https://unite.ut.ee/bl_forw.php?id=396271) | Rhodotorula | mucilaginosa | 1397 | 0.0 |
| [KF411548](https://unite.ut.ee/bl_forw.php?id=396273) | Rhodotorula | mucilaginosa | 1397 | 0.0 |
| [KF411547](https://unite.ut.ee/bl_forw.php?id=396274) | Rhodotorula | mucilaginosa | 1397 | 0.0 |
| [KF411544](https://unite.ut.ee/bl_forw.php?id=396277) | Rhodotorula | mucilaginosa | 1397 | 0.0 |
| [KF411543](https://unite.ut.ee/bl_forw.php?id=396278) | Rhodotorula | mucilaginosa | 1397 | 0.0 |
| [KF411537](https://unite.ut.ee/bl_forw.php?id=396284) | Rhodotorula | mucilaginosa | 1397 | 0.0 |
| [KF411536](https://unite.ut.ee/bl_forw.php?id=396285) | Rhodotorula | mucilaginosa | 1397 | 0.0 |
| [KF411533](https://unite.ut.ee/bl_forw.php?id=396288) | Rhodotorula | mucilaginosa | 1397 | 0.0 |
| [KF411530](https://unite.ut.ee/bl_forw.php?id=396291) | Rhodotorula | mucilaginosa | 1397 | 0.0 |
| [KF411528](https://unite.ut.ee/bl_forw.php?id=396293) | Rhodotorula | mucilaginosa | 1397 | 0.0 |
| [KF411525](https://unite.ut.ee/bl_forw.php?id=396296) | Rhodotorula | mucilaginosa | 1397 | 0.0 |
| [KF411524](https://unite.ut.ee/bl_forw.php?id=396297) | Rhodotorula | mucilaginosa | 1397 | 0.0 |
| [KF411523](https://unite.ut.ee/bl_forw.php?id=396298) | Rhodotorula | mucilaginosa | 1397 | 0.0 |
| [KF411522](https://unite.ut.ee/bl_forw.php?id=396299) | Rhodotorula | mucilaginosa | 1397 | 0.0 |
| [KF411521](https://unite.ut.ee/bl_forw.php?id=396300) | Rhodotorula | mucilaginosa | 1397 | 0.0 |
| [KF411518](https://unite.ut.ee/bl_forw.php?id=396303) | Rhodotorula | mucilaginosa | 1397 | 0.0 |
| [KF411517](https://unite.ut.ee/bl_forw.php?id=396304) | Rhodotorula | mucilaginosa | 1397 | 0.0 |
| [KF411515](https://unite.ut.ee/bl_forw.php?id=396306) | Rhodotorula | mucilaginosa | 1397 | 0.0 |
| [KF411512](https://unite.ut.ee/bl_forw.php?id=396309) | Rhodotorula | mucilaginosa | 1397 | 0.0 |
| [KF411503](https://unite.ut.ee/bl_forw.php?id=396318) | Rhodotorula | mucilaginosa | 1397 | 0.0 |
| [KF411501](https://unite.ut.ee/bl_forw.php?id=396320) | Rhodotorula | mucilaginosa | 1397 | 0.0 |
| [KF411494](https://unite.ut.ee/bl_forw.php?id=396327) | Rhodotorula | mucilaginosa | 1397 | 0.0 |
| [KF411488](https://unite.ut.ee/bl_forw.php?id=396333) | Rhodotorula | mucilaginosa | 1397 | 0.0 |
| [KF411486](https://unite.ut.ee/bl_forw.php?id=396335) | Rhodotorula | mucilaginosa | 1397 | 0.0 |
| [KF411484](https://unite.ut.ee/bl_forw.php?id=396337) | Rhodotorula | mucilaginosa | 1397 | 0.0 |
| [KF411481](https://unite.ut.ee/bl_forw.php?id=396340) | Rhodotorula | mucilaginosa | 1397 | 0.0 |
| [KF411475](https://unite.ut.ee/bl_forw.php?id=396346) | Rhodotorula | mucilaginosa | 1397 | 0.0 |
| [KF411472](https://unite.ut.ee/bl_forw.php?id=396349) | Rhodotorula | mucilaginosa | 1397 | 0.0 |
| [KF411468](https://unite.ut.ee/bl_forw.php?id=396353) | Rhodotorula | mucilaginosa | 1395 | 0.0 |

The 15 first alignments

**559 Rhodotorula mucilaginosa**

ngth=1136

2017-6-7 Blast output

Score = 1397 bits (1548), Expect = 0.0 Identities = 774/774 (100%), Gaps = 0/774 (0%) Strand=Plus/Plus

| Query | 1 | AGTGAATATAGGACGTCCAACTTAACTTGGAGTCCGAACTCTCACTTTCTAACCCTGTGC | 60 |
| --- | --- | --- | --- |
| Sbjct | 5 | \|\|\|\|\|\|\|\|\|\|\|\|\|\|\|\|\|\|\|\|\|\|\|\|\|\|\|\|\|\|\|\|\|\|\|\|\|\|\|\|\|\|\|\|\|\|\|\|\|\|\|\|\|\|\|\|\|\|\|\| AGTGAATATAGGACGTCCAACTTAACTTGGAGTCCGAACTCTCACTTTCTAACCCTGTGC | 64 |
| Query | 61 | ACTTGTTTGGGATAGTAACTCTCGCAAGAGAGCGAACTCCTATTCACTTATAAACACAAA | 120 |
| Sbjct | 65 | \|\|\|\|\|\|\|\|\|\|\|\|\|\|\|\|\|\|\|\|\|\|\|\|\|\|\|\|\|\|\|\|\|\|\|\|\|\|\|\|\|\|\|\|\|\|\|\|\|\|\|\|\|\|\|\|\|\|\|\| ACTTGTTTGGGATAGTAACTCTCGCAAGAGAGCGAACTCCTATTCACTTATAAACACAAA | 124 |
| Query | 121 | GTCTATGAATGTATTAAATTTTATAACAAAATAAAACTTTCAACAACGGATCTCTTGGCT | 180 |
| Sbjct | 125 | \|\|\|\|\|\|\|\|\|\|\|\|\|\|\|\|\|\|\|\|\|\|\|\|\|\|\|\|\|\|\|\|\|\|\|\|\|\|\|\|\|\|\|\|\|\|\|\|\|\|\|\|\|\|\|\|\|\|\|\| GTCTATGAATGTATTAAATTTTATAACAAAATAAAACTTTCAACAACGGATCTCTTGGCT | 184 |
| Query | 181 | CTCGCATCGATGAAGAACGCAGCGAAATGCGATAAGTAATGTGAATTGCAGAATTCAGTG | 240 |
| Sbjct | 185 | \|\|\|\|\|\|\|\|\|\|\|\|\|\|\|\|\|\|\|\|\|\|\|\|\|\|\|\|\|\|\|\|\|\|\|\|\|\|\|\|\|\|\|\|\|\|\|\|\|\|\|\|\|\|\|\|\|\|\|\| CTCGCATCGATGAAGAACGCAGCGAAATGCGATAAGTAATGTGAATTGCAGAATTCAGTG | 244 |
| Query | 241 | AATCATCGAATCTTTGAACGCACCTTGCGCTCCATGGTATTCCGTGGAGCATGCCTGTTT | 300 |
| Sbjct | 245 | \|\|\|\|\|\|\|\|\|\|\|\|\|\|\|\|\|\|\|\|\|\|\|\|\|\|\|\|\|\|\|\|\|\|\|\|\|\|\|\|\|\|\|\|\|\|\|\|\|\|\|\|\|\|\|\|\|\|\|\| AATCATCGAATCTTTGAACGCACCTTGCGCTCCATGGTATTCCGTGGAGCATGCCTGTTT | 304 |
| Query | 301 | GAGTGTCATGAATACTTCAACCCTCCTCTTTCTTAATGATTGAAGAGGTGTTTGGTTTCT | 360 |
| Sbjct | 305 | \|\|\|\|\|\|\|\|\|\|\|\|\|\|\|\|\|\|\|\|\|\|\|\|\|\|\|\|\|\|\|\|\|\|\|\|\|\|\|\|\|\|\|\|\|\|\|\|\|\|\|\|\|\|\|\|\|\|\|\| GAGTGTCATGAATACTTCAACCCTCCTCTTTCTTAATGATTGAAGAGGTGTTTGGTTTCT | 364 |
| Query | 361 | GAGCGCTGCTGGCCTTTACGGTCTAGCTCGTTCGTAATGCATTAGCATCCGCAATCGAAC | 420 |
| Sbjct | 365 | \|\|\|\|\|\|\|\|\|\|\|\|\|\|\|\|\|\|\|\|\|\|\|\|\|\|\|\|\|\|\|\|\|\|\|\|\|\|\|\|\|\|\|\|\|\|\|\|\|\|\|\|\|\|\|\|\|\|\|\| GAGCGCTGCTGGCCTTTACGGTCTAGCTCGTTCGTAATGCATTAGCATCCGCAATCGAAC | 424 |
| Query | 421 | TTCGGATTGACTTGGCGTAATAGACTATTCGCTGAGGAATTCTAGTCTTCGGATTAGAGC | 480 |
| Sbjct | 425 | \|\|\|\|\|\|\|\|\|\|\|\|\|\|\|\|\|\|\|\|\|\|\|\|\|\|\|\|\|\|\|\|\|\|\|\|\|\|\|\|\|\|\|\|\|\|\|\|\|\|\|\|\|\|\|\|\|\|\|\| TTCGGATTGACTTGGCGTAATAGACTATTCGCTGAGGAATTCTAGTCTTCGGATTAGAGC | 484 |
| Query | 481 | CGGGTTGGGTTAAAGGAAGCTTCTAATCAGAATGTCTACATTTTAAGATTAGATCTCAAA | 540 |
| Sbjct | 485 | \|\|\|\|\|\|\|\|\|\|\|\|\|\|\|\|\|\|\|\|\|\|\|\|\|\|\|\|\|\|\|\|\|\|\|\|\|\|\|\|\|\|\|\|\|\|\|\|\|\|\|\|\|\|\|\|\|\|\|\| CGGGTTGGGTTAAAGGAAGCTTCTAATCAGAATGTCTACATTTTAAGATTAGATCTCAAA | 544 |
| Query | 541 | TCAGGTAGGACTACCCGCTGAACTTAAGCATATCAATAAGCGGAGGAAAAGAAACTAACA | 600 |
| Sbjct | 545 | \|\|\|\|\|\|\|\|\|\|\|\|\|\|\|\|\|\|\|\|\|\|\|\|\|\|\|\|\|\|\|\|\|\|\|\|\|\|\|\|\|\|\|\|\|\|\|\|\|\|\|\|\|\|\|\|\|\|\|\| TCAGGTAGGACTACCCGCTGAACTTAAGCATATCAATAAGCGGAGGAAAAGAAACTAACA | 604 |
| Query | 601 | AGGATTCCCCTAGTAGCGGCGAGCGAAGCGGGAAGAGCTCAAATTTATAATCTGGCACCT | 660 |
| Sbjct | 605 | \|\|\|\|\|\|\|\|\|\|\|\|\|\|\|\|\|\|\|\|\|\|\|\|\|\|\|\|\|\|\|\|\|\|\|\|\|\|\|\|\|\|\|\|\|\|\|\|\|\|\|\|\|\|\|\|\|\|\|\| AGGATTCCCCTAGTAGCGGCGAGCGAAGCGGGAAGAGCTCAAATTTATAATCTGGCACCT | 664 |
| Query | 661 | TCGGTGTCCGAGTTGTAATCTCTAGAAATGTTTTCCGCGTTGGACCGCACACAAGTCTGT | 720 |
| Sbjct | 665 | \|\|\|\|\|\|\|\|\|\|\|\|\|\|\|\|\|\|\|\|\|\|\|\|\|\|\|\|\|\|\|\|\|\|\|\|\|\|\|\|\|\|\|\|\|\|\|\|\|\|\|\|\|\|\|\|\|\|\|\| TCGGTGTCCGAGTTGTAATCTCTAGAAATGTTTTCCGCGTTGGACCGCACACAAGTCTGT | 724 |
| Query  Sbjct | 721  725 | TGGAATACAGCGGCATAGTGGTGAGACCCCCGTATATGGTGCGGACGCCCAGCG 774  \|\|\|\|\|\|\|\|\|\|\|\|\|\|\|\|\|\|\|\|\|\|\|\|\|\|\|\|\|\|\|\|\|\|\|\|\|\|\|\|\|\|\|\|\|\|\|\|\|\|\|\|\|\| TGGAATACAGCGGCATAGTGGTGAGACCCCCGTATATGGTGCGGACGCCCAGCG 778 |  |

**> KF411558 Rhodotorula mucilaginosa**

Length=1132

Score = 1397 bits (1548), Expect = 0.0 Identities = 774/774 (100%), Gaps = 0/774 (0%)

Strand=Plus/Plus

| Query | 1 | AGTGAATATAGGACGTCCAACTTAACTTGGAGTCCGAACTCTCACTTTCTAACCCTGTGC | 60 |
| --- | --- | --- | --- |
| Sbjct | 4 | \|\|\|\|\|\|\|\|\|\|\|\|\|\|\|\|\|\|\|\|\|\|\|\|\|\|\|\|\|\|\|\|\|\|\|\|\|\|\|\|\|\|\|\|\|\|\|\|\|\|\|\|\|\|\|\|\|\|\|\| AGTGAATATAGGACGTCCAACTTAACTTGGAGTCCGAACTCTCACTTTCTAACCCTGTGC | 63 |
| Query | 61 | ACTTGTTTGGGATAGTAACTCTCGCAAGAGAGCGAACTCCTATTCACTTATAAACACAAA | 120 |
| Sbjct | 64 | \|\|\|\|\|\|\|\|\|\|\|\|\|\|\|\|\|\|\|\|\|\|\|\|\|\|\|\|\|\|\|\|\|\|\|\|\|\|\|\|\|\|\|\|\|\|\|\|\|\|\|\|\|\|\|\|\|\|\|\| ACTTGTTTGGGATAGTAACTCTCGCAAGAGAGCGAACTCCTATTCACTTATAAACACAAA | 123 |
| Query | 121 | GTCTATGAATGTATTAAATTTTATAACAAAATAAAACTTTCAACAACGGATCTCTTGGCT | 180 |
| Sbjct | 124 | \|\|\|\|\|\|\|\|\|\|\|\|\|\|\|\|\|\|\|\|\|\|\|\|\|\|\|\|\|\|\|\|\|\|\|\|\|\|\|\|\|\|\|\|\|\|\|\|\|\|\|\|\|\|\|\|\|\|\|\|  GTCTATGAATGTATTAAATTTTATAACAAAATAAAACTTTCAACAACGGATCTCTTGGCT | 183 |

https://unite.ut.ee/cgi-bin/seq_search3.cgi 2/14

| 2017-6-7  Query | 181 | Blast output  CTCGCATCGATGAAGAACGCAGCGAAATGCGATAAGTAATGTGAATTGCAGAATTCAGTG | 240 |
| --- | --- | --- | --- |
| Sbjct | 184 | \|\|\|\|\|\|\|\|\|\|\|\|\|\|\|\|\|\|\|\|\|\|\|\|\|\|\|\|\|\|\|\|\|\|\|\|\|\|\|\|\|\|\|\|\|\|\|\|\|\|\|\|\|\|\|\|\|\|\|\| CTCGCATCGATGAAGAACGCAGCGAAATGCGATAAGTAATGTGAATTGCAGAATTCAGTG | 243 |
| Query | 241 | AATCATCGAATCTTTGAACGCACCTTGCGCTCCATGGTATTCCGTGGAGCATGCCTGTTT | 300 |
| Sbjct | 244 | \|\|\|\|\|\|\|\|\|\|\|\|\|\|\|\|\|\|\|\|\|\|\|\|\|\|\|\|\|\|\|\|\|\|\|\|\|\|\|\|\|\|\|\|\|\|\|\|\|\|\|\|\|\|\|\|\|\|\|\| AATCATCGAATCTTTGAACGCACCTTGCGCTCCATGGTATTCCGTGGAGCATGCCTGTTT | 303 |
| Query | 301 | GAGTGTCATGAATACTTCAACCCTCCTCTTTCTTAATGATTGAAGAGGTGTTTGGTTTCT | 360 |
| Sbjct | 304 | \|\|\|\|\|\|\|\|\|\|\|\|\|\|\|\|\|\|\|\|\|\|\|\|\|\|\|\|\|\|\|\|\|\|\|\|\|\|\|\|\|\|\|\|\|\|\|\|\|\|\|\|\|\|\|\|\|\|\|\| GAGTGTCATGAATACTTCAACCCTCCTCTTTCTTAATGATTGAAGAGGTGTTTGGTTTCT | 363 |
| Query | 361 | GAGCGCTGCTGGCCTTTACGGTCTAGCTCGTTCGTAATGCATTAGCATCCGCAATCGAAC | 420 |
| Sbjct | 364 | \|\|\|\|\|\|\|\|\|\|\|\|\|\|\|\|\|\|\|\|\|\|\|\|\|\|\|\|\|\|\|\|\|\|\|\|\|\|\|\|\|\|\|\|\|\|\|\|\|\|\|\|\|\|\|\|\|\|\|\| GAGCGCTGCTGGCCTTTACGGTCTAGCTCGTTCGTAATGCATTAGCATCCGCAATCGAAC | 423 |
| Query | 421 | TTCGGATTGACTTGGCGTAATAGACTATTCGCTGAGGAATTCTAGTCTTCGGATTAGAGC | 480 |
| Sbjct | 424 | \|\|\|\|\|\|\|\|\|\|\|\|\|\|\|\|\|\|\|\|\|\|\|\|\|\|\|\|\|\|\|\|\|\|\|\|\|\|\|\|\|\|\|\|\|\|\|\|\|\|\|\|\|\|\|\|\|\|\|\| TTCGGATTGACTTGGCGTAATAGACTATTCGCTGAGGAATTCTAGTCTTCGGATTAGAGC | 483 |
| Query | 481 | CGGGTTGGGTTAAAGGAAGCTTCTAATCAGAATGTCTACATTTTAAGATTAGATCTCAAA | 540 |
| Sbjct | 484 | \|\|\|\|\|\|\|\|\|\|\|\|\|\|\|\|\|\|\|\|\|\|\|\|\|\|\|\|\|\|\|\|\|\|\|\|\|\|\|\|\|\|\|\|\|\|\|\|\|\|\|\|\|\|\|\|\|\|\|\| CGGGTTGGGTTAAAGGAAGCTTCTAATCAGAATGTCTACATTTTAAGATTAGATCTCAAA | 543 |
| Query | 541 | TCAGGTAGGACTACCCGCTGAACTTAAGCATATCAATAAGCGGAGGAAAAGAAACTAACA | 600 |
| Sbjct | 544 | \|\|\|\|\|\|\|\|\|\|\|\|\|\|\|\|\|\|\|\|\|\|\|\|\|\|\|\|\|\|\|\|\|\|\|\|\|\|\|\|\|\|\|\|\|\|\|\|\|\|\|\|\|\|\|\|\|\|\|\| TCAGGTAGGACTACCCGCTGAACTTAAGCATATCAATAAGCGGAGGAAAAGAAACTAACA | 603 |
| Query | 601 | AGGATTCCCCTAGTAGCGGCGAGCGAAGCGGGAAGAGCTCAAATTTATAATCTGGCACCT | 660 |
| Sbjct | 604 | \|\|\|\|\|\|\|\|\|\|\|\|\|\|\|\|\|\|\|\|\|\|\|\|\|\|\|\|\|\|\|\|\|\|\|\|\|\|\|\|\|\|\|\|\|\|\|\|\|\|\|\|\|\|\|\|\|\|\|\| AGGATTCCCCTAGTAGCGGCGAGCGAAGCGGGAAGAGCTCAAATTTATAATCTGGCACCT | 663 |
| Query | 661 | TCGGTGTCCGAGTTGTAATCTCTAGAAATGTTTTCCGCGTTGGACCGCACACAAGTCTGT | 720 |
| Sbjct | 664 | \|\|\|\|\|\|\|\|\|\|\|\|\|\|\|\|\|\|\|\|\|\|\|\|\|\|\|\|\|\|\|\|\|\|\|\|\|\|\|\|\|\|\|\|\|\|\|\|\|\|\|\|\|\|\|\|\|\|\|\| TCGGTGTCCGAGTTGTAATCTCTAGAAATGTTTTCCGCGTTGGACCGCACACAAGTCTGT | 723 |
| Query  Sbjct | 721  724 | TGGAATACAGCGGCATAGTGGTGAGACCCCCGTATATGGTGCGGACGCCCAGCG 774  \|\|\|\|\|\|\|\|\|\|\|\|\|\|\|\|\|\|\|\|\|\|\|\|\|\|\|\|\|\|\|\|\|\|\|\|\|\|\|\|\|\|\|\|\|\|\|\|\|\|\|\|\|\| TGGAATACAGCGGCATAGTGGTGAGACCCCCGTATATGGTGCGGACGCCCAGCG 777 |  |

**> KF411556 Rhodotorula mucilaginosa**

Length=1140

Score = 1397 bits (1548), Expect = 0.0 Identities = 774/774 (100%), Gaps = 0/774 (0%)

Strand=Plus/Plus

| Query | 1 | AGTGAATATAGGACGTCCAACTTAACTTGGAGTCCGAACTCTCACTTTCTAACCCTGTGC | 60 |
| --- | --- | --- | --- |
| Sbjct | 5 | \|\|\|\|\|\|\|\|\|\|\|\|\|\|\|\|\|\|\|\|\|\|\|\|\|\|\|\|\|\|\|\|\|\|\|\|\|\|\|\|\|\|\|\|\|\|\|\|\|\|\|\|\|\|\|\|\|\|\|\| AGTGAATATAGGACGTCCAACTTAACTTGGAGTCCGAACTCTCACTTTCTAACCCTGTGC | 64 |
| Query | 61 | ACTTGTTTGGGATAGTAACTCTCGCAAGAGAGCGAACTCCTATTCACTTATAAACACAAA | 120 |
| Sbjct | 65 | \|\|\|\|\|\|\|\|\|\|\|\|\|\|\|\|\|\|\|\|\|\|\|\|\|\|\|\|\|\|\|\|\|\|\|\|\|\|\|\|\|\|\|\|\|\|\|\|\|\|\|\|\|\|\|\|\|\|\|\| ACTTGTTTGGGATAGTAACTCTCGCAAGAGAGCGAACTCCTATTCACTTATAAACACAAA | 124 |
| Query | 121 | GTCTATGAATGTATTAAATTTTATAACAAAATAAAACTTTCAACAACGGATCTCTTGGCT | 180 |
| Sbjct | 125 | \|\|\|\|\|\|\|\|\|\|\|\|\|\|\|\|\|\|\|\|\|\|\|\|\|\|\|\|\|\|\|\|\|\|\|\|\|\|\|\|\|\|\|\|\|\|\|\|\|\|\|\|\|\|\|\|\|\|\|\| GTCTATGAATGTATTAAATTTTATAACAAAATAAAACTTTCAACAACGGATCTCTTGGCT | 184 |
| Query | 181 | CTCGCATCGATGAAGAACGCAGCGAAATGCGATAAGTAATGTGAATTGCAGAATTCAGTG | 240 |
| Sbjct | 185 | \|\|\|\|\|\|\|\|\|\|\|\|\|\|\|\|\|\|\|\|\|\|\|\|\|\|\|\|\|\|\|\|\|\|\|\|\|\|\|\|\|\|\|\|\|\|\|\|\|\|\|\|\|\|\|\|\|\|\|\| CTCGCATCGATGAAGAACGCAGCGAAATGCGATAAGTAATGTGAATTGCAGAATTCAGTG | 244 |
| Query | 241 | AATCATCGAATCTTTGAACGCACCTTGCGCTCCATGGTATTCCGTGGAGCATGCCTGTTT | 300 |
| Sbjct | 245 | \|\|\|\|\|\|\|\|\|\|\|\|\|\|\|\|\|\|\|\|\|\|\|\|\|\|\|\|\|\|\|\|\|\|\|\|\|\|\|\|\|\|\|\|\|\|\|\|\|\|\|\|\|\|\|\|\|\|\|\| AATCATCGAATCTTTGAACGCACCTTGCGCTCCATGGTATTCCGTGGAGCATGCCTGTTT | 304 |
| Query | 301 | GAGTGTCATGAATACTTCAACCCTCCTCTTTCTTAATGATTGAAGAGGTGTTTGGTTTCT | 360 |
| Sbjct | 305 | \|\|\|\|\|\|\|\|\|\|\|\|\|\|\|\|\|\|\|\|\|\|\|\|\|\|\|\|\|\|\|\|\|\|\|\|\|\|\|\|\|\|\|\|\|\|\|\|\|\|\|\|\|\|\|\|\|\|\|\| GAGTGTCATGAATACTTCAACCCTCCTCTTTCTTAATGATTGAAGAGGTGTTTGGTTTCT | 364 |
| Query | 361 | GAGCGCTGCTGGCCTTTACGGTCTAGCTCGTTCGTAATGCATTAGCATCCGCAATCGAAC | 420 |
| Sbjct | 365 | \|\|\|\|\|\|\|\|\|\|\|\|\|\|\|\|\|\|\|\|\|\|\|\|\|\|\|\|\|\|\|\|\|\|\|\|\|\|\|\|\|\|\|\|\|\|\|\|\|\|\|\|\|\|\|\|\|\|\|\|  GAGCGCTGCTGGCCTTTACGGTCTAGCTCGTTCGTAATGCATTAGCATCCGCAATCGAAC | 424 |

https://unite.ut.ee/cgi-bin/seq_search3.cgi 3/14

| 2017-6-7  Query | 421 | Blast output  TTCGGATTGACTTGGCGTAATAGACTATTCGCTGAGGAATTCTAGTCTTCGGATTAGAGC | 480 |
| --- | --- | --- | --- |
| Sbjct | 425 | \|\|\|\|\|\|\|\|\|\|\|\|\|\|\|\|\|\|\|\|\|\|\|\|\|\|\|\|\|\|\|\|\|\|\|\|\|\|\|\|\|\|\|\|\|\|\|\|\|\|\|\|\|\|\|\|\|\|\|\| TTCGGATTGACTTGGCGTAATAGACTATTCGCTGAGGAATTCTAGTCTTCGGATTAGAGC | 484 |
| Query | 481 | CGGGTTGGGTTAAAGGAAGCTTCTAATCAGAATGTCTACATTTTAAGATTAGATCTCAAA | 540 |
| Sbjct | 485 | \|\|\|\|\|\|\|\|\|\|\|\|\|\|\|\|\|\|\|\|\|\|\|\|\|\|\|\|\|\|\|\|\|\|\|\|\|\|\|\|\|\|\|\|\|\|\|\|\|\|\|\|\|\|\|\|\|\|\|\| CGGGTTGGGTTAAAGGAAGCTTCTAATCAGAATGTCTACATTTTAAGATTAGATCTCAAA | 544 |
| Query | 541 | TCAGGTAGGACTACCCGCTGAACTTAAGCATATCAATAAGCGGAGGAAAAGAAACTAACA | 600 |
| Sbjct | 545 | \|\|\|\|\|\|\|\|\|\|\|\|\|\|\|\|\|\|\|\|\|\|\|\|\|\|\|\|\|\|\|\|\|\|\|\|\|\|\|\|\|\|\|\|\|\|\|\|\|\|\|\|\|\|\|\|\|\|\|\| TCAGGTAGGACTACCCGCTGAACTTAAGCATATCAATAAGCGGAGGAAAAGAAACTAACA | 604 |
| Query | 601 | AGGATTCCCCTAGTAGCGGCGAGCGAAGCGGGAAGAGCTCAAATTTATAATCTGGCACCT | 660 |
| Sbjct | 605 | \|\|\|\|\|\|\|\|\|\|\|\|\|\|\|\|\|\|\|\|\|\|\|\|\|\|\|\|\|\|\|\|\|\|\|\|\|\|\|\|\|\|\|\|\|\|\|\|\|\|\|\|\|\|\|\|\|\|\|\| AGGATTCCCCTAGTAGCGGCGAGCGAAGCGGGAAGAGCTCAAATTTATAATCTGGCACCT | 664 |
| Query | 661 | TCGGTGTCCGAGTTGTAATCTCTAGAAATGTTTTCCGCGTTGGACCGCACACAAGTCTGT | 720 |
| Sbjct | 665 | \|\|\|\|\|\|\|\|\|\|\|\|\|\|\|\|\|\|\|\|\|\|\|\|\|\|\|\|\|\|\|\|\|\|\|\|\|\|\|\|\|\|\|\|\|\|\|\|\|\|\|\|\|\|\|\|\|\|\|\| TCGGTGTCCGAGTTGTAATCTCTAGAAATGTTTTCCGCGTTGGACCGCACACAAGTCTGT | 724 |
| Query  Sbjct | 721  725 | TGGAATACAGCGGCATAGTGGTGAGACCCCCGTATATGGTGCGGACGCCCAGCG 774  \|\|\|\|\|\|\|\|\|\|\|\|\|\|\|\|\|\|\|\|\|\|\|\|\|\|\|\|\|\|\|\|\|\|\|\|\|\|\|\|\|\|\|\|\|\|\|\|\|\|\|\|\|\| TGGAATACAGCGGCATAGTGGTGAGACCCCCGTATATGGTGCGGACGCCCAGCG 778 |  |

**> KF411555 Rhodotorula mucilaginosa**

Length=1141

Score = 1397 bits (1548), Expect = 0.0 Identities = 774/774 (100%), Gaps = 0/774 (0%)

Strand=Plus/Plus

| Query | 1 | AGTGAATATAGGACGTCCAACTTAACTTGGAGTCCGAACTCTCACTTTCTAACCCTGTGC | 60 |
| --- | --- | --- | --- |
| Sbjct | 12 | \|\|\|\|\|\|\|\|\|\|\|\|\|\|\|\|\|\|\|\|\|\|\|\|\|\|\|\|\|\|\|\|\|\|\|\|\|\|\|\|\|\|\|\|\|\|\|\|\|\|\|\|\|\|\|\|\|\|\|\| AGTGAATATAGGACGTCCAACTTAACTTGGAGTCCGAACTCTCACTTTCTAACCCTGTGC | 71 |
| Query | 61 | ACTTGTTTGGGATAGTAACTCTCGCAAGAGAGCGAACTCCTATTCACTTATAAACACAAA | 120 |
| Sbjct | 72 | \|\|\|\|\|\|\|\|\|\|\|\|\|\|\|\|\|\|\|\|\|\|\|\|\|\|\|\|\|\|\|\|\|\|\|\|\|\|\|\|\|\|\|\|\|\|\|\|\|\|\|\|\|\|\|\|\|\|\|\| ACTTGTTTGGGATAGTAACTCTCGCAAGAGAGCGAACTCCTATTCACTTATAAACACAAA | 131 |
| Query | 121 | GTCTATGAATGTATTAAATTTTATAACAAAATAAAACTTTCAACAACGGATCTCTTGGCT | 180 |
| Sbjct | 132 | \|\|\|\|\|\|\|\|\|\|\|\|\|\|\|\|\|\|\|\|\|\|\|\|\|\|\|\|\|\|\|\|\|\|\|\|\|\|\|\|\|\|\|\|\|\|\|\|\|\|\|\|\|\|\|\|\|\|\|\| GTCTATGAATGTATTAAATTTTATAACAAAATAAAACTTTCAACAACGGATCTCTTGGCT | 191 |
| Query | 181 | CTCGCATCGATGAAGAACGCAGCGAAATGCGATAAGTAATGTGAATTGCAGAATTCAGTG | 240 |
| Sbjct | 192 | \|\|\|\|\|\|\|\|\|\|\|\|\|\|\|\|\|\|\|\|\|\|\|\|\|\|\|\|\|\|\|\|\|\|\|\|\|\|\|\|\|\|\|\|\|\|\|\|\|\|\|\|\|\|\|\|\|\|\|\| CTCGCATCGATGAAGAACGCAGCGAAATGCGATAAGTAATGTGAATTGCAGAATTCAGTG | 251 |
| Query | 241 | AATCATCGAATCTTTGAACGCACCTTGCGCTCCATGGTATTCCGTGGAGCATGCCTGTTT | 300 |
| Sbjct | 252 | \|\|\|\|\|\|\|\|\|\|\|\|\|\|\|\|\|\|\|\|\|\|\|\|\|\|\|\|\|\|\|\|\|\|\|\|\|\|\|\|\|\|\|\|\|\|\|\|\|\|\|\|\|\|\|\|\|\|\|\| AATCATCGAATCTTTGAACGCACCTTGCGCTCCATGGTATTCCGTGGAGCATGCCTGTTT | 311 |
| Query | 301 | GAGTGTCATGAATACTTCAACCCTCCTCTTTCTTAATGATTGAAGAGGTGTTTGGTTTCT | 360 |
| Sbjct | 312 | \|\|\|\|\|\|\|\|\|\|\|\|\|\|\|\|\|\|\|\|\|\|\|\|\|\|\|\|\|\|\|\|\|\|\|\|\|\|\|\|\|\|\|\|\|\|\|\|\|\|\|\|\|\|\|\|\|\|\|\| GAGTGTCATGAATACTTCAACCCTCCTCTTTCTTAATGATTGAAGAGGTGTTTGGTTTCT | 371 |
| Query | 361 | GAGCGCTGCTGGCCTTTACGGTCTAGCTCGTTCGTAATGCATTAGCATCCGCAATCGAAC | 420 |
| Sbjct | 372 | \|\|\|\|\|\|\|\|\|\|\|\|\|\|\|\|\|\|\|\|\|\|\|\|\|\|\|\|\|\|\|\|\|\|\|\|\|\|\|\|\|\|\|\|\|\|\|\|\|\|\|\|\|\|\|\|\|\|\|\| GAGCGCTGCTGGCCTTTACGGTCTAGCTCGTTCGTAATGCATTAGCATCCGCAATCGAAC | 431 |
| Query | 421 | TTCGGATTGACTTGGCGTAATAGACTATTCGCTGAGGAATTCTAGTCTTCGGATTAGAGC | 480 |
| Sbjct | 432 | \|\|\|\|\|\|\|\|\|\|\|\|\|\|\|\|\|\|\|\|\|\|\|\|\|\|\|\|\|\|\|\|\|\|\|\|\|\|\|\|\|\|\|\|\|\|\|\|\|\|\|\|\|\|\|\|\|\|\|\| TTCGGATTGACTTGGCGTAATAGACTATTCGCTGAGGAATTCTAGTCTTCGGATTAGAGC | 491 |
| Query | 481 | CGGGTTGGGTTAAAGGAAGCTTCTAATCAGAATGTCTACATTTTAAGATTAGATCTCAAA | 540 |
| Sbjct | 492 | \|\|\|\|\|\|\|\|\|\|\|\|\|\|\|\|\|\|\|\|\|\|\|\|\|\|\|\|\|\|\|\|\|\|\|\|\|\|\|\|\|\|\|\|\|\|\|\|\|\|\|\|\|\|\|\|\|\|\|\| CGGGTTGGGTTAAAGGAAGCTTCTAATCAGAATGTCTACATTTTAAGATTAGATCTCAAA | 551 |
| Query | 541 | TCAGGTAGGACTACCCGCTGAACTTAAGCATATCAATAAGCGGAGGAAAAGAAACTAACA | 600 |
| Sbjct | 552 | \|\|\|\|\|\|\|\|\|\|\|\|\|\|\|\|\|\|\|\|\|\|\|\|\|\|\|\|\|\|\|\|\|\|\|\|\|\|\|\|\|\|\|\|\|\|\|\|\|\|\|\|\|\|\|\|\|\|\|\| TCAGGTAGGACTACCCGCTGAACTTAAGCATATCAATAAGCGGAGGAAAAGAAACTAACA | 611 |
| Query | 601 | AGGATTCCCCTAGTAGCGGCGAGCGAAGCGGGAAGAGCTCAAATTTATAATCTGGCACCT | 660 |
| Sbjct | 612 | \|\|\|\|\|\|\|\|\|\|\|\|\|\|\|\|\|\|\|\|\|\|\|\|\|\|\|\|\|\|\|\|\|\|\|\|\|\|\|\|\|\|\|\|\|\|\|\|\|\|\|\|\|\|\|\|\|\|\|\|  AGGATTCCCCTAGTAGCGGCGAGCGAAGCGGGAAGAGCTCAAATTTATAATCTGGCACCT | 671 |

https://unite.ut.ee/cgi-bin/seq_search3.cgi 4/14

| 2017-6-7  Query | Blast output  661 TCGGTGTCCGAGTTGTAATCTCTAGAAATGTTTTCCGCGTTGGACCGCACACAAGTCTGT | 720 |
| --- | --- | --- |
| Sbjct | \|\|\|\|\|\|\|\|\|\|\|\|\|\|\|\|\|\|\|\|\|\|\|\|\|\|\|\|\|\|\|\|\|\|\|\|\|\|\|\|\|\|\|\|\|\|\|\|\|\|\|\|\|\|\|\|\|\|\|\| 672 TCGGTGTCCGAGTTGTAATCTCTAGAAATGTTTTCCGCGTTGGACCGCACACAAGTCTGT | 731 |
| Query  Sbjct | 721 TGGAATACAGCGGCATAGTGGTGAGACCCCCGTATATGGTGCGGACGCCCAGCG 774  \|\|\|\|\|\|\|\|\|\|\|\|\|\|\|\|\|\|\|\|\|\|\|\|\|\|\|\|\|\|\|\|\|\|\|\|\|\|\|\|\|\|\|\|\|\|\|\|\|\|\|\|\|\|  732 TGGAATACAGCGGCATAGTGGTGAGACCCCCGTATATGGTGCGGACGCCCAGCG 785 |  |

**> KF411554 Rhodotorula mucilaginosa**

Length=1150

Score = 1397 bits (1548), Expect = 0.0 Identities = 774/774 (100%), Gaps = 0/774 (0%) Strand=Plus/Plus

| Query | 1 | AGTGAATATAGGACGTCCAACTTAACTTGGAGTCCGAACTCTCACTTTCTAACCCTGTGC | 60 |
| --- | --- | --- | --- |
| Sbjct | 12 | \|\|\|\|\|\|\|\|\|\|\|\|\|\|\|\|\|\|\|\|\|\|\|\|\|\|\|\|\|\|\|\|\|\|\|\|\|\|\|\|\|\|\|\|\|\|\|\|\|\|\|\|\|\|\|\|\|\|\|\| AGTGAATATAGGACGTCCAACTTAACTTGGAGTCCGAACTCTCACTTTCTAACCCTGTGC | 71 |
| Query | 61 | ACTTGTTTGGGATAGTAACTCTCGCAAGAGAGCGAACTCCTATTCACTTATAAACACAAA | 120 |
| Sbjct | 72 | \|\|\|\|\|\|\|\|\|\|\|\|\|\|\|\|\|\|\|\|\|\|\|\|\|\|\|\|\|\|\|\|\|\|\|\|\|\|\|\|\|\|\|\|\|\|\|\|\|\|\|\|\|\|\|\|\|\|\|\| ACTTGTTTGGGATAGTAACTCTCGCAAGAGAGCGAACTCCTATTCACTTATAAACACAAA | 131 |
| Query | 121 | GTCTATGAATGTATTAAATTTTATAACAAAATAAAACTTTCAACAACGGATCTCTTGGCT | 180 |
| Sbjct | 132 | \|\|\|\|\|\|\|\|\|\|\|\|\|\|\|\|\|\|\|\|\|\|\|\|\|\|\|\|\|\|\|\|\|\|\|\|\|\|\|\|\|\|\|\|\|\|\|\|\|\|\|\|\|\|\|\|\|\|\|\| GTCTATGAATGTATTAAATTTTATAACAAAATAAAACTTTCAACAACGGATCTCTTGGCT | 191 |
| Query | 181 | CTCGCATCGATGAAGAACGCAGCGAAATGCGATAAGTAATGTGAATTGCAGAATTCAGTG | 240 |
| Sbjct | 192 | \|\|\|\|\|\|\|\|\|\|\|\|\|\|\|\|\|\|\|\|\|\|\|\|\|\|\|\|\|\|\|\|\|\|\|\|\|\|\|\|\|\|\|\|\|\|\|\|\|\|\|\|\|\|\|\|\|\|\|\| CTCGCATCGATGAAGAACGCAGCGAAATGCGATAAGTAATGTGAATTGCAGAATTCAGTG | 251 |
| Query | 241 | AATCATCGAATCTTTGAACGCACCTTGCGCTCCATGGTATTCCGTGGAGCATGCCTGTTT | 300 |
| Sbjct | 252 | \|\|\|\|\|\|\|\|\|\|\|\|\|\|\|\|\|\|\|\|\|\|\|\|\|\|\|\|\|\|\|\|\|\|\|\|\|\|\|\|\|\|\|\|\|\|\|\|\|\|\|\|\|\|\|\|\|\|\|\| AATCATCGAATCTTTGAACGCACCTTGCGCTCCATGGTATTCCGTGGAGCATGCCTGTTT | 311 |
| Query | 301 | GAGTGTCATGAATACTTCAACCCTCCTCTTTCTTAATGATTGAAGAGGTGTTTGGTTTCT | 360 |
| Sbjct | 312 | \|\|\|\|\|\|\|\|\|\|\|\|\|\|\|\|\|\|\|\|\|\|\|\|\|\|\|\|\|\|\|\|\|\|\|\|\|\|\|\|\|\|\|\|\|\|\|\|\|\|\|\|\|\|\|\|\|\|\|\| GAGTGTCATGAATACTTCAACCCTCCTCTTTCTTAATGATTGAAGAGGTGTTTGGTTTCT | 371 |
| Query | 361 | GAGCGCTGCTGGCCTTTACGGTCTAGCTCGTTCGTAATGCATTAGCATCCGCAATCGAAC | 420 |
| Sbjct | 372 | \|\|\|\|\|\|\|\|\|\|\|\|\|\|\|\|\|\|\|\|\|\|\|\|\|\|\|\|\|\|\|\|\|\|\|\|\|\|\|\|\|\|\|\|\|\|\|\|\|\|\|\|\|\|\|\|\|\|\|\| GAGCGCTGCTGGCCTTTACGGTCTAGCTCGTTCGTAATGCATTAGCATCCGCAATCGAAC | 431 |
| Query | 421 | TTCGGATTGACTTGGCGTAATAGACTATTCGCTGAGGAATTCTAGTCTTCGGATTAGAGC | 480 |
| Sbjct | 432 | \|\|\|\|\|\|\|\|\|\|\|\|\|\|\|\|\|\|\|\|\|\|\|\|\|\|\|\|\|\|\|\|\|\|\|\|\|\|\|\|\|\|\|\|\|\|\|\|\|\|\|\|\|\|\|\|\|\|\|\| TTCGGATTGACTTGGCGTAATAGACTATTCGCTGAGGAATTCTAGTCTTCGGATTAGAGC | 491 |
| Query | 481 | CGGGTTGGGTTAAAGGAAGCTTCTAATCAGAATGTCTACATTTTAAGATTAGATCTCAAA | 540 |
| Sbjct | 492 | \|\|\|\|\|\|\|\|\|\|\|\|\|\|\|\|\|\|\|\|\|\|\|\|\|\|\|\|\|\|\|\|\|\|\|\|\|\|\|\|\|\|\|\|\|\|\|\|\|\|\|\|\|\|\|\|\|\|\|\| CGGGTTGGGTTAAAGGAAGCTTCTAATCAGAATGTCTACATTTTAAGATTAGATCTCAAA | 551 |
| Query | 541 | TCAGGTAGGACTACCCGCTGAACTTAAGCATATCAATAAGCGGAGGAAAAGAAACTAACA | 600 |
| Sbjct | 552 | \|\|\|\|\|\|\|\|\|\|\|\|\|\|\|\|\|\|\|\|\|\|\|\|\|\|\|\|\|\|\|\|\|\|\|\|\|\|\|\|\|\|\|\|\|\|\|\|\|\|\|\|\|\|\|\|\|\|\|\| TCAGGTAGGACTACCCGCTGAACTTAAGCATATCAATAAGCGGAGGAAAAGAAACTAACA | 611 |
| Query | 601 | AGGATTCCCCTAGTAGCGGCGAGCGAAGCGGGAAGAGCTCAAATTTATAATCTGGCACCT | 660 |
| Sbjct | 612 | \|\|\|\|\|\|\|\|\|\|\|\|\|\|\|\|\|\|\|\|\|\|\|\|\|\|\|\|\|\|\|\|\|\|\|\|\|\|\|\|\|\|\|\|\|\|\|\|\|\|\|\|\|\|\|\|\|\|\|\| AGGATTCCCCTAGTAGCGGCGAGCGAAGCGGGAAGAGCTCAAATTTATAATCTGGCACCT | 671 |
| Query | 661 | TCGGTGTCCGAGTTGTAATCTCTAGAAATGTTTTCCGCGTTGGACCGCACACAAGTCTGT | 720 |
| Sbjct | 672 | \|\|\|\|\|\|\|\|\|\|\|\|\|\|\|\|\|\|\|\|\|\|\|\|\|\|\|\|\|\|\|\|\|\|\|\|\|\|\|\|\|\|\|\|\|\|\|\|\|\|\|\|\|\|\|\|\|\|\|\| TCGGTGTCCGAGTTGTAATCTCTAGAAATGTTTTCCGCGTTGGACCGCACACAAGTCTGT | 731 |
| Query  Sbjct | 721  732 | TGGAATACAGCGGCATAGTGGTGAGACCCCCGTATATGGTGCGGACGCCCAGCG 774  \|\|\|\|\|\|\|\|\|\|\|\|\|\|\|\|\|\|\|\|\|\|\|\|\|\|\|\|\|\|\|\|\|\|\|\|\|\|\|\|\|\|\|\|\|\|\|\|\|\|\|\|\|\| TGGAATACAGCGGCATAGTGGTGAGACCCCCGTATATGGTGCGGACGCCCAGCG 785 |  |

**> KF411553 Rhodotorula mucilaginosa**

Length=1139

https://unite.ut.ee/cgi-bin/seq_search3.cgi 5/14

2017-6-7 Blast output

Score = 1397 bits (1548), Expect = 0.0 Identities = 774/774 (100%), Gaps = 0/774 (0%) Strand=Plus/Plus

| Query | 1 | AGTGAATATAGGACGTCCAACTTAACTTGGAGTCCGAACTCTCACTTTCTAACCCTGTGC | 60 |
| --- | --- | --- | --- |
| Sbjct | 5 | \|\|\|\|\|\|\|\|\|\|\|\|\|\|\|\|\|\|\|\|\|\|\|\|\|\|\|\|\|\|\|\|\|\|\|\|\|\|\|\|\|\|\|\|\|\|\|\|\|\|\|\|\|\|\|\|\|\|\|\| AGTGAATATAGGACGTCCAACTTAACTTGGAGTCCGAACTCTCACTTTCTAACCCTGTGC | 64 |
| Query | 61 | ACTTGTTTGGGATAGTAACTCTCGCAAGAGAGCGAACTCCTATTCACTTATAAACACAAA | 120 |
| Sbjct | 65 | \|\|\|\|\|\|\|\|\|\|\|\|\|\|\|\|\|\|\|\|\|\|\|\|\|\|\|\|\|\|\|\|\|\|\|\|\|\|\|\|\|\|\|\|\|\|\|\|\|\|\|\|\|\|\|\|\|\|\|\| ACTTGTTTGGGATAGTAACTCTCGCAAGAGAGCGAACTCCTATTCACTTATAAACACAAA | 124 |
| Query | 121 | GTCTATGAATGTATTAAATTTTATAACAAAATAAAACTTTCAACAACGGATCTCTTGGCT | 180 |
| Sbjct | 125 | \|\|\|\|\|\|\|\|\|\|\|\|\|\|\|\|\|\|\|\|\|\|\|\|\|\|\|\|\|\|\|\|\|\|\|\|\|\|\|\|\|\|\|\|\|\|\|\|\|\|\|\|\|\|\|\|\|\|\|\| GTCTATGAATGTATTAAATTTTATAACAAAATAAAACTTTCAACAACGGATCTCTTGGCT | 184 |
| Query | 181 | CTCGCATCGATGAAGAACGCAGCGAAATGCGATAAGTAATGTGAATTGCAGAATTCAGTG | 240 |
| Sbjct | 185 | \|\|\|\|\|\|\|\|\|\|\|\|\|\|\|\|\|\|\|\|\|\|\|\|\|\|\|\|\|\|\|\|\|\|\|\|\|\|\|\|\|\|\|\|\|\|\|\|\|\|\|\|\|\|\|\|\|\|\|\| CTCGCATCGATGAAGAACGCAGCGAAATGCGATAAGTAATGTGAATTGCAGAATTCAGTG | 244 |
| Query | 241 | AATCATCGAATCTTTGAACGCACCTTGCGCTCCATGGTATTCCGTGGAGCATGCCTGTTT | 300 |
| Sbjct | 245 | \|\|\|\|\|\|\|\|\|\|\|\|\|\|\|\|\|\|\|\|\|\|\|\|\|\|\|\|\|\|\|\|\|\|\|\|\|\|\|\|\|\|\|\|\|\|\|\|\|\|\|\|\|\|\|\|\|\|\|\| AATCATCGAATCTTTGAACGCACCTTGCGCTCCATGGTATTCCGTGGAGCATGCCTGTTT | 304 |
| Query | 301 | GAGTGTCATGAATACTTCAACCCTCCTCTTTCTTAATGATTGAAGAGGTGTTTGGTTTCT | 360 |
| Sbjct | 305 | \|\|\|\|\|\|\|\|\|\|\|\|\|\|\|\|\|\|\|\|\|\|\|\|\|\|\|\|\|\|\|\|\|\|\|\|\|\|\|\|\|\|\|\|\|\|\|\|\|\|\|\|\|\|\|\|\|\|\|\| GAGTGTCATGAATACTTCAACCCTCCTCTTTCTTAATGATTGAAGAGGTGTTTGGTTTCT | 364 |
| Query | 361 | GAGCGCTGCTGGCCTTTACGGTCTAGCTCGTTCGTAATGCATTAGCATCCGCAATCGAAC | 420 |
| Sbjct | 365 | \|\|\|\|\|\|\|\|\|\|\|\|\|\|\|\|\|\|\|\|\|\|\|\|\|\|\|\|\|\|\|\|\|\|\|\|\|\|\|\|\|\|\|\|\|\|\|\|\|\|\|\|\|\|\|\|\|\|\|\| GAGCGCTGCTGGCCTTTACGGTCTAGCTCGTTCGTAATGCATTAGCATCCGCAATCGAAC | 424 |
| Query | 421 | TTCGGATTGACTTGGCGTAATAGACTATTCGCTGAGGAATTCTAGTCTTCGGATTAGAGC | 480 |
| Sbjct | 425 | \|\|\|\|\|\|\|\|\|\|\|\|\|\|\|\|\|\|\|\|\|\|\|\|\|\|\|\|\|\|\|\|\|\|\|\|\|\|\|\|\|\|\|\|\|\|\|\|\|\|\|\|\|\|\|\|\|\|\|\| TTCGGATTGACTTGGCGTAATAGACTATTCGCTGAGGAATTCTAGTCTTCGGATTAGAGC | 484 |
| Query | 481 | CGGGTTGGGTTAAAGGAAGCTTCTAATCAGAATGTCTACATTTTAAGATTAGATCTCAAA | 540 |
| Sbjct | 485 | \|\|\|\|\|\|\|\|\|\|\|\|\|\|\|\|\|\|\|\|\|\|\|\|\|\|\|\|\|\|\|\|\|\|\|\|\|\|\|\|\|\|\|\|\|\|\|\|\|\|\|\|\|\|\|\|\|\|\|\| CGGGTTGGGTTAAAGGAAGCTTCTAATCAGAATGTCTACATTTTAAGATTAGATCTCAAA | 544 |
| Query | 541 | TCAGGTAGGACTACCCGCTGAACTTAAGCATATCAATAAGCGGAGGAAAAGAAACTAACA | 600 |
| Sbjct | 545 | \|\|\|\|\|\|\|\|\|\|\|\|\|\|\|\|\|\|\|\|\|\|\|\|\|\|\|\|\|\|\|\|\|\|\|\|\|\|\|\|\|\|\|\|\|\|\|\|\|\|\|\|\|\|\|\|\|\|\|\| TCAGGTAGGACTACCCGCTGAACTTAAGCATATCAATAAGCGGAGGAAAAGAAACTAACA | 604 |
| Query | 601 | AGGATTCCCCTAGTAGCGGCGAGCGAAGCGGGAAGAGCTCAAATTTATAATCTGGCACCT | 660 |
| Sbjct | 605 | \|\|\|\|\|\|\|\|\|\|\|\|\|\|\|\|\|\|\|\|\|\|\|\|\|\|\|\|\|\|\|\|\|\|\|\|\|\|\|\|\|\|\|\|\|\|\|\|\|\|\|\|\|\|\|\|\|\|\|\| AGGATTCCCCTAGTAGCGGCGAGCGAAGCGGGAAGAGCTCAAATTTATAATCTGGCACCT | 664 |
| Query | 661 | TCGGTGTCCGAGTTGTAATCTCTAGAAATGTTTTCCGCGTTGGACCGCACACAAGTCTGT | 720 |
| Sbjct | 665 | \|\|\|\|\|\|\|\|\|\|\|\|\|\|\|\|\|\|\|\|\|\|\|\|\|\|\|\|\|\|\|\|\|\|\|\|\|\|\|\|\|\|\|\|\|\|\|\|\|\|\|\|\|\|\|\|\|\|\|\| TCGGTGTCCGAGTTGTAATCTCTAGAAATGTTTTCCGCGTTGGACCGCACACAAGTCTGT | 724 |
| Query  Sbjct | 721  725 | TGGAATACAGCGGCATAGTGGTGAGACCCCCGTATATGGTGCGGACGCCCAGCG 774  \|\|\|\|\|\|\|\|\|\|\|\|\|\|\|\|\|\|\|\|\|\|\|\|\|\|\|\|\|\|\|\|\|\|\|\|\|\|\|\|\|\|\|\|\|\|\|\|\|\|\|\|\|\| TGGAATACAGCGGCATAGTGGTGAGACCCCCGTATATGGTGCGGACGCCCAGCG 778 |  |

**> KF411552 Rhodotorula mucilaginosa**

Length=1150

Score = 1397 bits (1548), Expect = 0.0 Identities = 774/774 (100%), Gaps = 0/774 (0%)

Strand=Plus/Plus

| Query | 1 | AGTGAATATAGGACGTCCAACTTAACTTGGAGTCCGAACTCTCACTTTCTAACCCTGTGC | 60 |
| --- | --- | --- | --- |
| Sbjct | 13 | \|\|\|\|\|\|\|\|\|\|\|\|\|\|\|\|\|\|\|\|\|\|\|\|\|\|\|\|\|\|\|\|\|\|\|\|\|\|\|\|\|\|\|\|\|\|\|\|\|\|\|\|\|\|\|\|\|\|\|\| AGTGAATATAGGACGTCCAACTTAACTTGGAGTCCGAACTCTCACTTTCTAACCCTGTGC | 72 |
| Query | 61 | ACTTGTTTGGGATAGTAACTCTCGCAAGAGAGCGAACTCCTATTCACTTATAAACACAAA | 120 |
| Sbjct | 73 | \|\|\|\|\|\|\|\|\|\|\|\|\|\|\|\|\|\|\|\|\|\|\|\|\|\|\|\|\|\|\|\|\|\|\|\|\|\|\|\|\|\|\|\|\|\|\|\|\|\|\|\|\|\|\|\|\|\|\|\| ACTTGTTTGGGATAGTAACTCTCGCAAGAGAGCGAACTCCTATTCACTTATAAACACAAA | 132 |
| Query | 121 | GTCTATGAATGTATTAAATTTTATAACAAAATAAAACTTTCAACAACGGATCTCTTGGCT | 180 |
| Sbjct | 133 | \|\|\|\|\|\|\|\|\|\|\|\|\|\|\|\|\|\|\|\|\|\|\|\|\|\|\|\|\|\|\|\|\|\|\|\|\|\|\|\|\|\|\|\|\|\|\|\|\|\|\|\|\|\|\|\|\|\|\|\|  GTCTATGAATGTATTAAATTTTATAACAAAATAAAACTTTCAACAACGGATCTCTTGGCT | 192 |

https://unite.ut.ee/cgi-bin/seq_search3.cgi 6/14

| 2017-6-7  Query | 181 | Blast output  CTCGCATCGATGAAGAACGCAGCGAAATGCGATAAGTAATGTGAATTGCAGAATTCAGTG | 240 |
| --- | --- | --- | --- |
| Sbjct | 193 | \|\|\|\|\|\|\|\|\|\|\|\|\|\|\|\|\|\|\|\|\|\|\|\|\|\|\|\|\|\|\|\|\|\|\|\|\|\|\|\|\|\|\|\|\|\|\|\|\|\|\|\|\|\|\|\|\|\|\|\| CTCGCATCGATGAAGAACGCAGCGAAATGCGATAAGTAATGTGAATTGCAGAATTCAGTG | 252 |
| Query | 241 | AATCATCGAATCTTTGAACGCACCTTGCGCTCCATGGTATTCCGTGGAGCATGCCTGTTT | 300 |
| Sbjct | 253 | \|\|\|\|\|\|\|\|\|\|\|\|\|\|\|\|\|\|\|\|\|\|\|\|\|\|\|\|\|\|\|\|\|\|\|\|\|\|\|\|\|\|\|\|\|\|\|\|\|\|\|\|\|\|\|\|\|\|\|\| AATCATCGAATCTTTGAACGCACCTTGCGCTCCATGGTATTCCGTGGAGCATGCCTGTTT | 312 |
| Query | 301 | GAGTGTCATGAATACTTCAACCCTCCTCTTTCTTAATGATTGAAGAGGTGTTTGGTTTCT | 360 |
| Sbjct | 313 | \|\|\|\|\|\|\|\|\|\|\|\|\|\|\|\|\|\|\|\|\|\|\|\|\|\|\|\|\|\|\|\|\|\|\|\|\|\|\|\|\|\|\|\|\|\|\|\|\|\|\|\|\|\|\|\|\|\|\|\| GAGTGTCATGAATACTTCAACCCTCCTCTTTCTTAATGATTGAAGAGGTGTTTGGTTTCT | 372 |
| Query | 361 | GAGCGCTGCTGGCCTTTACGGTCTAGCTCGTTCGTAATGCATTAGCATCCGCAATCGAAC | 420 |
| Sbjct | 373 | \|\|\|\|\|\|\|\|\|\|\|\|\|\|\|\|\|\|\|\|\|\|\|\|\|\|\|\|\|\|\|\|\|\|\|\|\|\|\|\|\|\|\|\|\|\|\|\|\|\|\|\|\|\|\|\|\|\|\|\| GAGCGCTGCTGGCCTTTACGGTCTAGCTCGTTCGTAATGCATTAGCATCCGCAATCGAAC | 432 |
| Query | 421 | TTCGGATTGACTTGGCGTAATAGACTATTCGCTGAGGAATTCTAGTCTTCGGATTAGAGC | 480 |
| Sbjct | 433 | \|\|\|\|\|\|\|\|\|\|\|\|\|\|\|\|\|\|\|\|\|\|\|\|\|\|\|\|\|\|\|\|\|\|\|\|\|\|\|\|\|\|\|\|\|\|\|\|\|\|\|\|\|\|\|\|\|\|\|\| TTCGGATTGACTTGGCGTAATAGACTATTCGCTGAGGAATTCTAGTCTTCGGATTAGAGC | 492 |
| Query | 481 | CGGGTTGGGTTAAAGGAAGCTTCTAATCAGAATGTCTACATTTTAAGATTAGATCTCAAA | 540 |
| Sbjct | 493 | \|\|\|\|\|\|\|\|\|\|\|\|\|\|\|\|\|\|\|\|\|\|\|\|\|\|\|\|\|\|\|\|\|\|\|\|\|\|\|\|\|\|\|\|\|\|\|\|\|\|\|\|\|\|\|\|\|\|\|\| CGGGTTGGGTTAAAGGAAGCTTCTAATCAGAATGTCTACATTTTAAGATTAGATCTCAAA | 552 |
| Query | 541 | TCAGGTAGGACTACCCGCTGAACTTAAGCATATCAATAAGCGGAGGAAAAGAAACTAACA | 600 |
| Sbjct | 553 | \|\|\|\|\|\|\|\|\|\|\|\|\|\|\|\|\|\|\|\|\|\|\|\|\|\|\|\|\|\|\|\|\|\|\|\|\|\|\|\|\|\|\|\|\|\|\|\|\|\|\|\|\|\|\|\|\|\|\|\| TCAGGTAGGACTACCCGCTGAACTTAAGCATATCAATAAGCGGAGGAAAAGAAACTAACA | 612 |
| Query | 601 | AGGATTCCCCTAGTAGCGGCGAGCGAAGCGGGAAGAGCTCAAATTTATAATCTGGCACCT | 660 |
| Sbjct | 613 | \|\|\|\|\|\|\|\|\|\|\|\|\|\|\|\|\|\|\|\|\|\|\|\|\|\|\|\|\|\|\|\|\|\|\|\|\|\|\|\|\|\|\|\|\|\|\|\|\|\|\|\|\|\|\|\|\|\|\|\| AGGATTCCCCTAGTAGCGGCGAGCGAAGCGGGAAGAGCTCAAATTTATAATCTGGCACCT | 672 |
| Query | 661 | TCGGTGTCCGAGTTGTAATCTCTAGAAATGTTTTCCGCGTTGGACCGCACACAAGTCTGT | 720 |
| Sbjct | 673 | \|\|\|\|\|\|\|\|\|\|\|\|\|\|\|\|\|\|\|\|\|\|\|\|\|\|\|\|\|\|\|\|\|\|\|\|\|\|\|\|\|\|\|\|\|\|\|\|\|\|\|\|\|\|\|\|\|\|\|\| TCGGTGTCCGAGTTGTAATCTCTAGAAATGTTTTCCGCGTTGGACCGCACACAAGTCTGT | 732 |
| Query  Sbjct | 721  733 | TGGAATACAGCGGCATAGTGGTGAGACCCCCGTATATGGTGCGGACGCCCAGCG 774  \|\|\|\|\|\|\|\|\|\|\|\|\|\|\|\|\|\|\|\|\|\|\|\|\|\|\|\|\|\|\|\|\|\|\|\|\|\|\|\|\|\|\|\|\|\|\|\|\|\|\|\|\|\| TGGAATACAGCGGCATAGTGGTGAGACCCCCGTATATGGTGCGGACGCCCAGCG 786 |  |

**> KF411551 Rhodotorula mucilaginosa**

Length=1141

Score = 1397 bits (1548), Expect = 0.0 Identities = 774/774 (100%), Gaps = 0/774 (0%)

Strand=Plus/Plus

| Query | 1 | AGTGAATATAGGACGTCCAACTTAACTTGGAGTCCGAACTCTCACTTTCTAACCCTGTGC | 60 |
| --- | --- | --- | --- |
| Sbjct | 8 | \|\|\|\|\|\|\|\|\|\|\|\|\|\|\|\|\|\|\|\|\|\|\|\|\|\|\|\|\|\|\|\|\|\|\|\|\|\|\|\|\|\|\|\|\|\|\|\|\|\|\|\|\|\|\|\|\|\|\|\| AGTGAATATAGGACGTCCAACTTAACTTGGAGTCCGAACTCTCACTTTCTAACCCTGTGC | 67 |
| Query | 61 | ACTTGTTTGGGATAGTAACTCTCGCAAGAGAGCGAACTCCTATTCACTTATAAACACAAA | 120 |
| Sbjct | 68 | \|\|\|\|\|\|\|\|\|\|\|\|\|\|\|\|\|\|\|\|\|\|\|\|\|\|\|\|\|\|\|\|\|\|\|\|\|\|\|\|\|\|\|\|\|\|\|\|\|\|\|\|\|\|\|\|\|\|\|\| ACTTGTTTGGGATAGTAACTCTCGCAAGAGAGCGAACTCCTATTCACTTATAAACACAAA | 127 |
| Query | 121 | GTCTATGAATGTATTAAATTTTATAACAAAATAAAACTTTCAACAACGGATCTCTTGGCT | 180 |
| Sbjct | 128 | \|\|\|\|\|\|\|\|\|\|\|\|\|\|\|\|\|\|\|\|\|\|\|\|\|\|\|\|\|\|\|\|\|\|\|\|\|\|\|\|\|\|\|\|\|\|\|\|\|\|\|\|\|\|\|\|\|\|\|\| GTCTATGAATGTATTAAATTTTATAACAAAATAAAACTTTCAACAACGGATCTCTTGGCT | 187 |
| Query | 181 | CTCGCATCGATGAAGAACGCAGCGAAATGCGATAAGTAATGTGAATTGCAGAATTCAGTG | 240 |
| Sbjct | 188 | \|\|\|\|\|\|\|\|\|\|\|\|\|\|\|\|\|\|\|\|\|\|\|\|\|\|\|\|\|\|\|\|\|\|\|\|\|\|\|\|\|\|\|\|\|\|\|\|\|\|\|\|\|\|\|\|\|\|\|\| CTCGCATCGATGAAGAACGCAGCGAAATGCGATAAGTAATGTGAATTGCAGAATTCAGTG | 247 |
| Query | 241 | AATCATCGAATCTTTGAACGCACCTTGCGCTCCATGGTATTCCGTGGAGCATGCCTGTTT | 300 |
| Sbjct | 248 | \|\|\|\|\|\|\|\|\|\|\|\|\|\|\|\|\|\|\|\|\|\|\|\|\|\|\|\|\|\|\|\|\|\|\|\|\|\|\|\|\|\|\|\|\|\|\|\|\|\|\|\|\|\|\|\|\|\|\|\| AATCATCGAATCTTTGAACGCACCTTGCGCTCCATGGTATTCCGTGGAGCATGCCTGTTT | 307 |
| Query | 301 | GAGTGTCATGAATACTTCAACCCTCCTCTTTCTTAATGATTGAAGAGGTGTTTGGTTTCT | 360 |
| Sbjct | 308 | \|\|\|\|\|\|\|\|\|\|\|\|\|\|\|\|\|\|\|\|\|\|\|\|\|\|\|\|\|\|\|\|\|\|\|\|\|\|\|\|\|\|\|\|\|\|\|\|\|\|\|\|\|\|\|\|\|\|\|\| GAGTGTCATGAATACTTCAACCCTCCTCTTTCTTAATGATTGAAGAGGTGTTTGGTTTCT | 367 |
| Query | 361 | GAGCGCTGCTGGCCTTTACGGTCTAGCTCGTTCGTAATGCATTAGCATCCGCAATCGAAC | 420 |
| Sbjct | 368 | \|\|\|\|\|\|\|\|\|\|\|\|\|\|\|\|\|\|\|\|\|\|\|\|\|\|\|\|\|\|\|\|\|\|\|\|\|\|\|\|\|\|\|\|\|\|\|\|\|\|\|\|\|\|\|\|\|\|\|\|  GAGCGCTGCTGGCCTTTACGGTCTAGCTCGTTCGTAATGCATTAGCATCCGCAATCGAAC | 427 |

https://unite.ut.ee/cgi-bin/seq_search3.cgi 7/14

| 2017-6-7  Query | 421 | Blast output  TTCGGATTGACTTGGCGTAATAGACTATTCGCTGAGGAATTCTAGTCTTCGGATTAGAGC | 480 |
| --- | --- | --- | --- |
| Sbjct | 428 | \|\|\|\|\|\|\|\|\|\|\|\|\|\|\|\|\|\|\|\|\|\|\|\|\|\|\|\|\|\|\|\|\|\|\|\|\|\|\|\|\|\|\|\|\|\|\|\|\|\|\|\|\|\|\|\|\|\|\|\| TTCGGATTGACTTGGCGTAATAGACTATTCGCTGAGGAATTCTAGTCTTCGGATTAGAGC | 487 |
| Query | 481 | CGGGTTGGGTTAAAGGAAGCTTCTAATCAGAATGTCTACATTTTAAGATTAGATCTCAAA | 540 |
| Sbjct | 488 | \|\|\|\|\|\|\|\|\|\|\|\|\|\|\|\|\|\|\|\|\|\|\|\|\|\|\|\|\|\|\|\|\|\|\|\|\|\|\|\|\|\|\|\|\|\|\|\|\|\|\|\|\|\|\|\|\|\|\|\| CGGGTTGGGTTAAAGGAAGCTTCTAATCAGAATGTCTACATTTTAAGATTAGATCTCAAA | 547 |
| Query | 541 | TCAGGTAGGACTACCCGCTGAACTTAAGCATATCAATAAGCGGAGGAAAAGAAACTAACA | 600 |
| Sbjct | 548 | \|\|\|\|\|\|\|\|\|\|\|\|\|\|\|\|\|\|\|\|\|\|\|\|\|\|\|\|\|\|\|\|\|\|\|\|\|\|\|\|\|\|\|\|\|\|\|\|\|\|\|\|\|\|\|\|\|\|\|\| TCAGGTAGGACTACCCGCTGAACTTAAGCATATCAATAAGCGGAGGAAAAGAAACTAACA | 607 |
| Query | 601 | AGGATTCCCCTAGTAGCGGCGAGCGAAGCGGGAAGAGCTCAAATTTATAATCTGGCACCT | 660 |
| Sbjct | 608 | \|\|\|\|\|\|\|\|\|\|\|\|\|\|\|\|\|\|\|\|\|\|\|\|\|\|\|\|\|\|\|\|\|\|\|\|\|\|\|\|\|\|\|\|\|\|\|\|\|\|\|\|\|\|\|\|\|\|\|\| AGGATTCCCCTAGTAGCGGCGAGCGAAGCGGGAAGAGCTCAAATTTATAATCTGGCACCT | 667 |
| Query | 661 | TCGGTGTCCGAGTTGTAATCTCTAGAAATGTTTTCCGCGTTGGACCGCACACAAGTCTGT | 720 |
| Sbjct | 668 | \|\|\|\|\|\|\|\|\|\|\|\|\|\|\|\|\|\|\|\|\|\|\|\|\|\|\|\|\|\|\|\|\|\|\|\|\|\|\|\|\|\|\|\|\|\|\|\|\|\|\|\|\|\|\|\|\|\|\|\| TCGGTGTCCGAGTTGTAATCTCTAGAAATGTTTTCCGCGTTGGACCGCACACAAGTCTGT | 727 |
| Query  Sbjct | 721  728 | TGGAATACAGCGGCATAGTGGTGAGACCCCCGTATATGGTGCGGACGCCCAGCG 774  \|\|\|\|\|\|\|\|\|\|\|\|\|\|\|\|\|\|\|\|\|\|\|\|\|\|\|\|\|\|\|\|\|\|\|\|\|\|\|\|\|\|\|\|\|\|\|\|\|\|\|\|\|\| TGGAATACAGCGGCATAGTGGTGAGACCCCCGTATATGGTGCGGACGCCCAGCG 781 |  |

**> KF411550 Rhodotorula mucilaginosa**

Length=1131

Score = 1397 bits (1548), Expect = 0.0 Identities = 774/774 (100%), Gaps = 0/774 (0%)

Strand=Plus/Plus

| Query | 1 | AGTGAATATAGGACGTCCAACTTAACTTGGAGTCCGAACTCTCACTTTCTAACCCTGTGC | 60 |
| --- | --- | --- | --- |
| Sbjct | 2 | \|\|\|\|\|\|\|\|\|\|\|\|\|\|\|\|\|\|\|\|\|\|\|\|\|\|\|\|\|\|\|\|\|\|\|\|\|\|\|\|\|\|\|\|\|\|\|\|\|\|\|\|\|\|\|\|\|\|\|\| AGTGAATATAGGACGTCCAACTTAACTTGGAGTCCGAACTCTCACTTTCTAACCCTGTGC | 61 |
| Query | 61 | ACTTGTTTGGGATAGTAACTCTCGCAAGAGAGCGAACTCCTATTCACTTATAAACACAAA | 120 |
| Sbjct | 62 | \|\|\|\|\|\|\|\|\|\|\|\|\|\|\|\|\|\|\|\|\|\|\|\|\|\|\|\|\|\|\|\|\|\|\|\|\|\|\|\|\|\|\|\|\|\|\|\|\|\|\|\|\|\|\|\|\|\|\|\| ACTTGTTTGGGATAGTAACTCTCGCAAGAGAGCGAACTCCTATTCACTTATAAACACAAA | 121 |
| Query | 121 | GTCTATGAATGTATTAAATTTTATAACAAAATAAAACTTTCAACAACGGATCTCTTGGCT | 180 |
| Sbjct | 122 | \|\|\|\|\|\|\|\|\|\|\|\|\|\|\|\|\|\|\|\|\|\|\|\|\|\|\|\|\|\|\|\|\|\|\|\|\|\|\|\|\|\|\|\|\|\|\|\|\|\|\|\|\|\|\|\|\|\|\|\| GTCTATGAATGTATTAAATTTTATAACAAAATAAAACTTTCAACAACGGATCTCTTGGCT | 181 |
| Query | 181 | CTCGCATCGATGAAGAACGCAGCGAAATGCGATAAGTAATGTGAATTGCAGAATTCAGTG | 240 |
| Sbjct | 182 | \|\|\|\|\|\|\|\|\|\|\|\|\|\|\|\|\|\|\|\|\|\|\|\|\|\|\|\|\|\|\|\|\|\|\|\|\|\|\|\|\|\|\|\|\|\|\|\|\|\|\|\|\|\|\|\|\|\|\|\| CTCGCATCGATGAAGAACGCAGCGAAATGCGATAAGTAATGTGAATTGCAGAATTCAGTG | 241 |
| Query | 241 | AATCATCGAATCTTTGAACGCACCTTGCGCTCCATGGTATTCCGTGGAGCATGCCTGTTT | 300 |
| Sbjct | 242 | \|\|\|\|\|\|\|\|\|\|\|\|\|\|\|\|\|\|\|\|\|\|\|\|\|\|\|\|\|\|\|\|\|\|\|\|\|\|\|\|\|\|\|\|\|\|\|\|\|\|\|\|\|\|\|\|\|\|\|\| AATCATCGAATCTTTGAACGCACCTTGCGCTCCATGGTATTCCGTGGAGCATGCCTGTTT | 301 |
| Query | 301 | GAGTGTCATGAATACTTCAACCCTCCTCTTTCTTAATGATTGAAGAGGTGTTTGGTTTCT | 360 |
| Sbjct | 302 | \|\|\|\|\|\|\|\|\|\|\|\|\|\|\|\|\|\|\|\|\|\|\|\|\|\|\|\|\|\|\|\|\|\|\|\|\|\|\|\|\|\|\|\|\|\|\|\|\|\|\|\|\|\|\|\|\|\|\|\| GAGTGTCATGAATACTTCAACCCTCCTCTTTCTTAATGATTGAAGAGGTGTTTGGTTTCT | 361 |
| Query | 361 | GAGCGCTGCTGGCCTTTACGGTCTAGCTCGTTCGTAATGCATTAGCATCCGCAATCGAAC | 420 |
| Sbjct | 362 | \|\|\|\|\|\|\|\|\|\|\|\|\|\|\|\|\|\|\|\|\|\|\|\|\|\|\|\|\|\|\|\|\|\|\|\|\|\|\|\|\|\|\|\|\|\|\|\|\|\|\|\|\|\|\|\|\|\|\|\| GAGCGCTGCTGGCCTTTACGGTCTAGCTCGTTCGTAATGCATTAGCATCCGCAATCGAAC | 421 |
| Query | 421 | TTCGGATTGACTTGGCGTAATAGACTATTCGCTGAGGAATTCTAGTCTTCGGATTAGAGC | 480 |
| Sbjct | 422 | \|\|\|\|\|\|\|\|\|\|\|\|\|\|\|\|\|\|\|\|\|\|\|\|\|\|\|\|\|\|\|\|\|\|\|\|\|\|\|\|\|\|\|\|\|\|\|\|\|\|\|\|\|\|\|\|\|\|\|\| TTCGGATTGACTTGGCGTAATAGACTATTCGCTGAGGAATTCTAGTCTTCGGATTAGAGC | 481 |
| Query | 481 | CGGGTTGGGTTAAAGGAAGCTTCTAATCAGAATGTCTACATTTTAAGATTAGATCTCAAA | 540 |
| Sbjct | 482 | \|\|\|\|\|\|\|\|\|\|\|\|\|\|\|\|\|\|\|\|\|\|\|\|\|\|\|\|\|\|\|\|\|\|\|\|\|\|\|\|\|\|\|\|\|\|\|\|\|\|\|\|\|\|\|\|\|\|\|\| CGGGTTGGGTTAAAGGAAGCTTCTAATCAGAATGTCTACATTTTAAGATTAGATCTCAAA | 541 |
| Query | 541 | TCAGGTAGGACTACCCGCTGAACTTAAGCATATCAATAAGCGGAGGAAAAGAAACTAACA | 600 |
| Sbjct | 542 | \|\|\|\|\|\|\|\|\|\|\|\|\|\|\|\|\|\|\|\|\|\|\|\|\|\|\|\|\|\|\|\|\|\|\|\|\|\|\|\|\|\|\|\|\|\|\|\|\|\|\|\|\|\|\|\|\|\|\|\| TCAGGTAGGACTACCCGCTGAACTTAAGCATATCAATAAGCGGAGGAAAAGAAACTAACA | 601 |
| Query | 601 | AGGATTCCCCTAGTAGCGGCGAGCGAAGCGGGAAGAGCTCAAATTTATAATCTGGCACCT | 660 |
| Sbjct | 602 | \|\|\|\|\|\|\|\|\|\|\|\|\|\|\|\|\|\|\|\|\|\|\|\|\|\|\|\|\|\|\|\|\|\|\|\|\|\|\|\|\|\|\|\|\|\|\|\|\|\|\|\|\|\|\|\|\|\|\|\|  AGGATTCCCCTAGTAGCGGCGAGCGAAGCGGGAAGAGCTCAAATTTATAATCTGGCACCT | 661 |

https://unite.ut.ee/cgi-bin/seq_search3.cgi 8/14

2017-6-7 Blast output

| Query | 661 TCGGTGTCCGAGTTGTAATCTCTAGAAATGTTTTCCGCGTTGGACCGCACACAAGTCTGT | 720 |
| --- | --- | --- |
|  | \|\|\|\|\|\|\|\|\|\|\|\|\|\|\|\|\|\|\|\|\|\|\|\|\|\|\|\|\|\|\|\|\|\|\|\|\|\|\|\|\|\|\|\|\|\|\|\|\|\|\|\|\|\|\|\|\|\|\|\| |  |
| Sbjct | 662 TCGGTGTCCGAGTTGTAATCTCTAGAAATGTTTTCCGCGTTGGACCGCACACAAGTCTGT | 721 |

Query 721 TGGAATACAGCGGCATAGTGGTGAGACCCCCGTATATGGTGCGGACGCCCAGCG 774

|||||||||||||||||||||||||||||||||||||||||||||||||||||| Sbjct 722 TGGAATACAGCGGCATAGTGGTGAGACCCCCGTATATGGTGCGGACGCCCAGCG 775

**> KF411548 Rhodotorula mucilaginosa**

Length=1130

Score = 1397 bits (1548), Expect = 0.0 Identities = 774/774 (100%), Gaps = 0/774 (0%) Strand=Plus/Plus

| Query | 1 | AGTGAATATAGGACGTCCAACTTAACTTGGAGTCCGAACTCTCACTTTCTAACCCTGTGC | 60 |
| --- | --- | --- | --- |
| Sbjct | 2 | \|\|\|\|\|\|\|\|\|\|\|\|\|\|\|\|\|\|\|\|\|\|\|\|\|\|\|\|\|\|\|\|\|\|\|\|\|\|\|\|\|\|\|\|\|\|\|\|\|\|\|\|\|\|\|\|\|\|\|\| AGTGAATATAGGACGTCCAACTTAACTTGGAGTCCGAACTCTCACTTTCTAACCCTGTGC | 61 |
| Query | 61 | ACTTGTTTGGGATAGTAACTCTCGCAAGAGAGCGAACTCCTATTCACTTATAAACACAAA | 120 |
| Sbjct | 62 | \|\|\|\|\|\|\|\|\|\|\|\|\|\|\|\|\|\|\|\|\|\|\|\|\|\|\|\|\|\|\|\|\|\|\|\|\|\|\|\|\|\|\|\|\|\|\|\|\|\|\|\|\|\|\|\|\|\|\|\| ACTTGTTTGGGATAGTAACTCTCGCAAGAGAGCGAACTCCTATTCACTTATAAACACAAA | 121 |
| Query | 121 | GTCTATGAATGTATTAAATTTTATAACAAAATAAAACTTTCAACAACGGATCTCTTGGCT | 180 |
| Sbjct | 122 | \|\|\|\|\|\|\|\|\|\|\|\|\|\|\|\|\|\|\|\|\|\|\|\|\|\|\|\|\|\|\|\|\|\|\|\|\|\|\|\|\|\|\|\|\|\|\|\|\|\|\|\|\|\|\|\|\|\|\|\| GTCTATGAATGTATTAAATTTTATAACAAAATAAAACTTTCAACAACGGATCTCTTGGCT | 181 |
| Query | 181 | CTCGCATCGATGAAGAACGCAGCGAAATGCGATAAGTAATGTGAATTGCAGAATTCAGTG | 240 |
| Sbjct | 182 | \|\|\|\|\|\|\|\|\|\|\|\|\|\|\|\|\|\|\|\|\|\|\|\|\|\|\|\|\|\|\|\|\|\|\|\|\|\|\|\|\|\|\|\|\|\|\|\|\|\|\|\|\|\|\|\|\|\|\|\| CTCGCATCGATGAAGAACGCAGCGAAATGCGATAAGTAATGTGAATTGCAGAATTCAGTG | 241 |
| Query | 241 | AATCATCGAATCTTTGAACGCACCTTGCGCTCCATGGTATTCCGTGGAGCATGCCTGTTT | 300 |
| Sbjct | 242 | \|\|\|\|\|\|\|\|\|\|\|\|\|\|\|\|\|\|\|\|\|\|\|\|\|\|\|\|\|\|\|\|\|\|\|\|\|\|\|\|\|\|\|\|\|\|\|\|\|\|\|\|\|\|\|\|\|\|\|\| AATCATCGAATCTTTGAACGCACCTTGCGCTCCATGGTATTCCGTGGAGCATGCCTGTTT | 301 |
| Query | 301 | GAGTGTCATGAATACTTCAACCCTCCTCTTTCTTAATGATTGAAGAGGTGTTTGGTTTCT | 360 |
| Sbjct | 302 | \|\|\|\|\|\|\|\|\|\|\|\|\|\|\|\|\|\|\|\|\|\|\|\|\|\|\|\|\|\|\|\|\|\|\|\|\|\|\|\|\|\|\|\|\|\|\|\|\|\|\|\|\|\|\|\|\|\|\|\| GAGTGTCATGAATACTTCAACCCTCCTCTTTCTTAATGATTGAAGAGGTGTTTGGTTTCT | 361 |
| Query | 361 | GAGCGCTGCTGGCCTTTACGGTCTAGCTCGTTCGTAATGCATTAGCATCCGCAATCGAAC | 420 |
| Sbjct | 362 | \|\|\|\|\|\|\|\|\|\|\|\|\|\|\|\|\|\|\|\|\|\|\|\|\|\|\|\|\|\|\|\|\|\|\|\|\|\|\|\|\|\|\|\|\|\|\|\|\|\|\|\|\|\|\|\|\|\|\|\| GAGCGCTGCTGGCCTTTACGGTCTAGCTCGTTCGTAATGCATTAGCATCCGCAATCGAAC | 421 |
| Query | 421 | TTCGGATTGACTTGGCGTAATAGACTATTCGCTGAGGAATTCTAGTCTTCGGATTAGAGC | 480 |
| Sbjct | 422 | \|\|\|\|\|\|\|\|\|\|\|\|\|\|\|\|\|\|\|\|\|\|\|\|\|\|\|\|\|\|\|\|\|\|\|\|\|\|\|\|\|\|\|\|\|\|\|\|\|\|\|\|\|\|\|\|\|\|\|\| TTCGGATTGACTTGGCGTAATAGACTATTCGCTGAGGAATTCTAGTCTTCGGATTAGAGC | 481 |
| Query | 481 | CGGGTTGGGTTAAAGGAAGCTTCTAATCAGAATGTCTACATTTTAAGATTAGATCTCAAA | 540 |
| Sbjct | 482 | \|\|\|\|\|\|\|\|\|\|\|\|\|\|\|\|\|\|\|\|\|\|\|\|\|\|\|\|\|\|\|\|\|\|\|\|\|\|\|\|\|\|\|\|\|\|\|\|\|\|\|\|\|\|\|\|\|\|\|\| CGGGTTGGGTTAAAGGAAGCTTCTAATCAGAATGTCTACATTTTAAGATTAGATCTCAAA | 541 |
| Query | 541 | TCAGGTAGGACTACCCGCTGAACTTAAGCATATCAATAAGCGGAGGAAAAGAAACTAACA | 600 |
| Sbjct | 542 | \|\|\|\|\|\|\|\|\|\|\|\|\|\|\|\|\|\|\|\|\|\|\|\|\|\|\|\|\|\|\|\|\|\|\|\|\|\|\|\|\|\|\|\|\|\|\|\|\|\|\|\|\|\|\|\|\|\|\|\| TCAGGTAGGACTACCCGCTGAACTTAAGCATATCAATAAGCGGAGGAAAAGAAACTAACA | 601 |
| Query | 601 | AGGATTCCCCTAGTAGCGGCGAGCGAAGCGGGAAGAGCTCAAATTTATAATCTGGCACCT | 660 |
| Sbjct | 602 | \|\|\|\|\|\|\|\|\|\|\|\|\|\|\|\|\|\|\|\|\|\|\|\|\|\|\|\|\|\|\|\|\|\|\|\|\|\|\|\|\|\|\|\|\|\|\|\|\|\|\|\|\|\|\|\|\|\|\|\| AGGATTCCCCTAGTAGCGGCGAGCGAAGCGGGAAGAGCTCAAATTTATAATCTGGCACCT | 661 |
| Query | 661 | TCGGTGTCCGAGTTGTAATCTCTAGAAATGTTTTCCGCGTTGGACCGCACACAAGTCTGT | 720 |
| Sbjct | 662 | \|\|\|\|\|\|\|\|\|\|\|\|\|\|\|\|\|\|\|\|\|\|\|\|\|\|\|\|\|\|\|\|\|\|\|\|\|\|\|\|\|\|\|\|\|\|\|\|\|\|\|\|\|\|\|\|\|\|\|\| TCGGTGTCCGAGTTGTAATCTCTAGAAATGTTTTCCGCGTTGGACCGCACACAAGTCTGT | 721 |
| Query  Sbjct | 721  722 | TGGAATACAGCGGCATAGTGGTGAGACCCCCGTATATGGTGCGGACGCCCAGCG 774  \|\|\|\|\|\|\|\|\|\|\|\|\|\|\|\|\|\|\|\|\|\|\|\|\|\|\|\|\|\|\|\|\|\|\|\|\|\|\|\|\|\|\|\|\|\|\|\|\|\|\|\|\|\| TGGAATACAGCGGCATAGTGGTGAGACCCCCGTATATGGTGCGGACGCCCAGCG 775 |  |

**> KF411547 Rhodotorula mucilaginosa**

Length=1133

https://unite.ut.ee/cgi-bin/seq_search3.cgi 9/14

2017-6-7 Blast output

Score = 1397 bits (1548), Expect = 0.0 Identities = 774/774 (100%), Gaps = 0/774 (0%) Strand=Plus/Plus

| Query | 1 | AGTGAATATAGGACGTCCAACTTAACTTGGAGTCCGAACTCTCACTTTCTAACCCTGTGC | 60 |
| --- | --- | --- | --- |
| Sbjct | 3 | \|\|\|\|\|\|\|\|\|\|\|\|\|\|\|\|\|\|\|\|\|\|\|\|\|\|\|\|\|\|\|\|\|\|\|\|\|\|\|\|\|\|\|\|\|\|\|\|\|\|\|\|\|\|\|\|\|\|\|\| AGTGAATATAGGACGTCCAACTTAACTTGGAGTCCGAACTCTCACTTTCTAACCCTGTGC | 62 |
| Query | 61 | ACTTGTTTGGGATAGTAACTCTCGCAAGAGAGCGAACTCCTATTCACTTATAAACACAAA | 120 |
| Sbjct | 63 | \|\|\|\|\|\|\|\|\|\|\|\|\|\|\|\|\|\|\|\|\|\|\|\|\|\|\|\|\|\|\|\|\|\|\|\|\|\|\|\|\|\|\|\|\|\|\|\|\|\|\|\|\|\|\|\|\|\|\|\| ACTTGTTTGGGATAGTAACTCTCGCAAGAGAGCGAACTCCTATTCACTTATAAACACAAA | 122 |
| Query | 121 | GTCTATGAATGTATTAAATTTTATAACAAAATAAAACTTTCAACAACGGATCTCTTGGCT | 180 |
| Sbjct | 123 | \|\|\|\|\|\|\|\|\|\|\|\|\|\|\|\|\|\|\|\|\|\|\|\|\|\|\|\|\|\|\|\|\|\|\|\|\|\|\|\|\|\|\|\|\|\|\|\|\|\|\|\|\|\|\|\|\|\|\|\| GTCTATGAATGTATTAAATTTTATAACAAAATAAAACTTTCAACAACGGATCTCTTGGCT | 182 |
| Query | 181 | CTCGCATCGATGAAGAACGCAGCGAAATGCGATAAGTAATGTGAATTGCAGAATTCAGTG | 240 |
| Sbjct | 183 | \|\|\|\|\|\|\|\|\|\|\|\|\|\|\|\|\|\|\|\|\|\|\|\|\|\|\|\|\|\|\|\|\|\|\|\|\|\|\|\|\|\|\|\|\|\|\|\|\|\|\|\|\|\|\|\|\|\|\|\| CTCGCATCGATGAAGAACGCAGCGAAATGCGATAAGTAATGTGAATTGCAGAATTCAGTG | 242 |
| Query | 241 | AATCATCGAATCTTTGAACGCACCTTGCGCTCCATGGTATTCCGTGGAGCATGCCTGTTT | 300 |
| Sbjct | 243 | \|\|\|\|\|\|\|\|\|\|\|\|\|\|\|\|\|\|\|\|\|\|\|\|\|\|\|\|\|\|\|\|\|\|\|\|\|\|\|\|\|\|\|\|\|\|\|\|\|\|\|\|\|\|\|\|\|\|\|\| AATCATCGAATCTTTGAACGCACCTTGCGCTCCATGGTATTCCGTGGAGCATGCCTGTTT | 302 |
| Query | 301 | GAGTGTCATGAATACTTCAACCCTCCTCTTTCTTAATGATTGAAGAGGTGTTTGGTTTCT | 360 |
| Sbjct | 303 | \|\|\|\|\|\|\|\|\|\|\|\|\|\|\|\|\|\|\|\|\|\|\|\|\|\|\|\|\|\|\|\|\|\|\|\|\|\|\|\|\|\|\|\|\|\|\|\|\|\|\|\|\|\|\|\|\|\|\|\| GAGTGTCATGAATACTTCAACCCTCCTCTTTCTTAATGATTGAAGAGGTGTTTGGTTTCT | 362 |
| Query | 361 | GAGCGCTGCTGGCCTTTACGGTCTAGCTCGTTCGTAATGCATTAGCATCCGCAATCGAAC | 420 |
| Sbjct | 363 | \|\|\|\|\|\|\|\|\|\|\|\|\|\|\|\|\|\|\|\|\|\|\|\|\|\|\|\|\|\|\|\|\|\|\|\|\|\|\|\|\|\|\|\|\|\|\|\|\|\|\|\|\|\|\|\|\|\|\|\| GAGCGCTGCTGGCCTTTACGGTCTAGCTCGTTCGTAATGCATTAGCATCCGCAATCGAAC | 422 |
| Query | 421 | TTCGGATTGACTTGGCGTAATAGACTATTCGCTGAGGAATTCTAGTCTTCGGATTAGAGC | 480 |
| Sbjct | 423 | \|\|\|\|\|\|\|\|\|\|\|\|\|\|\|\|\|\|\|\|\|\|\|\|\|\|\|\|\|\|\|\|\|\|\|\|\|\|\|\|\|\|\|\|\|\|\|\|\|\|\|\|\|\|\|\|\|\|\|\| TTCGGATTGACTTGGCGTAATAGACTATTCGCTGAGGAATTCTAGTCTTCGGATTAGAGC | 482 |
| Query | 481 | CGGGTTGGGTTAAAGGAAGCTTCTAATCAGAATGTCTACATTTTAAGATTAGATCTCAAA | 540 |
| Sbjct | 483 | \|\|\|\|\|\|\|\|\|\|\|\|\|\|\|\|\|\|\|\|\|\|\|\|\|\|\|\|\|\|\|\|\|\|\|\|\|\|\|\|\|\|\|\|\|\|\|\|\|\|\|\|\|\|\|\|\|\|\|\| CGGGTTGGGTTAAAGGAAGCTTCTAATCAGAATGTCTACATTTTAAGATTAGATCTCAAA | 542 |
| Query | 541 | TCAGGTAGGACTACCCGCTGAACTTAAGCATATCAATAAGCGGAGGAAAAGAAACTAACA | 600 |
| Sbjct | 543 | \|\|\|\|\|\|\|\|\|\|\|\|\|\|\|\|\|\|\|\|\|\|\|\|\|\|\|\|\|\|\|\|\|\|\|\|\|\|\|\|\|\|\|\|\|\|\|\|\|\|\|\|\|\|\|\|\|\|\|\| TCAGGTAGGACTACCCGCTGAACTTAAGCATATCAATAAGCGGAGGAAAAGAAACTAACA | 602 |
| Query | 601 | AGGATTCCCCTAGTAGCGGCGAGCGAAGCGGGAAGAGCTCAAATTTATAATCTGGCACCT | 660 |
| Sbjct | 603 | \|\|\|\|\|\|\|\|\|\|\|\|\|\|\|\|\|\|\|\|\|\|\|\|\|\|\|\|\|\|\|\|\|\|\|\|\|\|\|\|\|\|\|\|\|\|\|\|\|\|\|\|\|\|\|\|\|\|\|\| AGGATTCCCCTAGTAGCGGCGAGCGAAGCGGGAAGAGCTCAAATTTATAATCTGGCACCT | 662 |
| Query | 661 | TCGGTGTCCGAGTTGTAATCTCTAGAAATGTTTTCCGCGTTGGACCGCACACAAGTCTGT | 720 |
| Sbjct | 663 | \|\|\|\|\|\|\|\|\|\|\|\|\|\|\|\|\|\|\|\|\|\|\|\|\|\|\|\|\|\|\|\|\|\|\|\|\|\|\|\|\|\|\|\|\|\|\|\|\|\|\|\|\|\|\|\|\|\|\|\| TCGGTGTCCGAGTTGTAATCTCTAGAAATGTTTTCCGCGTTGGACCGCACACAAGTCTGT | 722 |
| Query  Sbjct | 721  723 | TGGAATACAGCGGCATAGTGGTGAGACCCCCGTATATGGTGCGGACGCCCAGCG 774  \|\|\|\|\|\|\|\|\|\|\|\|\|\|\|\|\|\|\|\|\|\|\|\|\|\|\|\|\|\|\|\|\|\|\|\|\|\|\|\|\|\|\|\|\|\|\|\|\|\|\|\|\|\| TGGAATACAGCGGCATAGTGGTGAGACCCCCGTATATGGTGCGGACGCCCAGCG 776 |  |

**> KF411544 Rhodotorula mucilaginosa**

Length=1129

Score = 1397 bits (1548), Expect = 0.0 Identities = 774/774 (100%), Gaps = 0/774 (0%)

Strand=Plus/Plus

| Query | 1 | AGTGAATATAGGACGTCCAACTTAACTTGGAGTCCGAACTCTCACTTTCTAACCCTGTGC | 60 |
| --- | --- | --- | --- |
| Sbjct | 2 | \|\|\|\|\|\|\|\|\|\|\|\|\|\|\|\|\|\|\|\|\|\|\|\|\|\|\|\|\|\|\|\|\|\|\|\|\|\|\|\|\|\|\|\|\|\|\|\|\|\|\|\|\|\|\|\|\|\|\|\| AGTGAATATAGGACGTCCAACTTAACTTGGAGTCCGAACTCTCACTTTCTAACCCTGTGC | 61 |
| Query | 61 | ACTTGTTTGGGATAGTAACTCTCGCAAGAGAGCGAACTCCTATTCACTTATAAACACAAA | 120 |
| Sbjct | 62 | \|\|\|\|\|\|\|\|\|\|\|\|\|\|\|\|\|\|\|\|\|\|\|\|\|\|\|\|\|\|\|\|\|\|\|\|\|\|\|\|\|\|\|\|\|\|\|\|\|\|\|\|\|\|\|\|\|\|\|\| ACTTGTTTGGGATAGTAACTCTCGCAAGAGAGCGAACTCCTATTCACTTATAAACACAAA | 121 |
| Query | 121 | GTCTATGAATGTATTAAATTTTATAACAAAATAAAACTTTCAACAACGGATCTCTTGGCT | 180 |
| Sbjct | 122 | \|\|\|\|\|\|\|\|\|\|\|\|\|\|\|\|\|\|\|\|\|\|\|\|\|\|\|\|\|\|\|\|\|\|\|\|\|\|\|\|\|\|\|\|\|\|\|\|\|\|\|\|\|\|\|\|\|\|\|\|  GTCTATGAATGTATTAAATTTTATAACAAAATAAAACTTTCAACAACGGATCTCTTGGCT | 181 |

https://unite.ut.ee/cgi-bin/seq_search3.cgi 10/14

| 2017-6-7  Query | 181 | Blast output  CTCGCATCGATGAAGAACGCAGCGAAATGCGATAAGTAATGTGAATTGCAGAATTCAGTG | 240 |
| --- | --- | --- | --- |
| Sbjct | 182 | \|\|\|\|\|\|\|\|\|\|\|\|\|\|\|\|\|\|\|\|\|\|\|\|\|\|\|\|\|\|\|\|\|\|\|\|\|\|\|\|\|\|\|\|\|\|\|\|\|\|\|\|\|\|\|\|\|\|\|\| CTCGCATCGATGAAGAACGCAGCGAAATGCGATAAGTAATGTGAATTGCAGAATTCAGTG | 241 |
| Query | 241 | AATCATCGAATCTTTGAACGCACCTTGCGCTCCATGGTATTCCGTGGAGCATGCCTGTTT | 300 |
| Sbjct | 242 | \|\|\|\|\|\|\|\|\|\|\|\|\|\|\|\|\|\|\|\|\|\|\|\|\|\|\|\|\|\|\|\|\|\|\|\|\|\|\|\|\|\|\|\|\|\|\|\|\|\|\|\|\|\|\|\|\|\|\|\| AATCATCGAATCTTTGAACGCACCTTGCGCTCCATGGTATTCCGTGGAGCATGCCTGTTT | 301 |
| Query | 301 | GAGTGTCATGAATACTTCAACCCTCCTCTTTCTTAATGATTGAAGAGGTGTTTGGTTTCT | 360 |
| Sbjct | 302 | \|\|\|\|\|\|\|\|\|\|\|\|\|\|\|\|\|\|\|\|\|\|\|\|\|\|\|\|\|\|\|\|\|\|\|\|\|\|\|\|\|\|\|\|\|\|\|\|\|\|\|\|\|\|\|\|\|\|\|\| GAGTGTCATGAATACTTCAACCCTCCTCTTTCTTAATGATTGAAGAGGTGTTTGGTTTCT | 361 |
| Query | 361 | GAGCGCTGCTGGCCTTTACGGTCTAGCTCGTTCGTAATGCATTAGCATCCGCAATCGAAC | 420 |
| Sbjct | 362 | \|\|\|\|\|\|\|\|\|\|\|\|\|\|\|\|\|\|\|\|\|\|\|\|\|\|\|\|\|\|\|\|\|\|\|\|\|\|\|\|\|\|\|\|\|\|\|\|\|\|\|\|\|\|\|\|\|\|\|\| GAGCGCTGCTGGCCTTTACGGTCTAGCTCGTTCGTAATGCATTAGCATCCGCAATCGAAC | 421 |
| Query | 421 | TTCGGATTGACTTGGCGTAATAGACTATTCGCTGAGGAATTCTAGTCTTCGGATTAGAGC | 480 |
| Sbjct | 422 | \|\|\|\|\|\|\|\|\|\|\|\|\|\|\|\|\|\|\|\|\|\|\|\|\|\|\|\|\|\|\|\|\|\|\|\|\|\|\|\|\|\|\|\|\|\|\|\|\|\|\|\|\|\|\|\|\|\|\|\| TTCGGATTGACTTGGCGTAATAGACTATTCGCTGAGGAATTCTAGTCTTCGGATTAGAGC | 481 |
| Query | 481 | CGGGTTGGGTTAAAGGAAGCTTCTAATCAGAATGTCTACATTTTAAGATTAGATCTCAAA | 540 |
| Sbjct | 482 | \|\|\|\|\|\|\|\|\|\|\|\|\|\|\|\|\|\|\|\|\|\|\|\|\|\|\|\|\|\|\|\|\|\|\|\|\|\|\|\|\|\|\|\|\|\|\|\|\|\|\|\|\|\|\|\|\|\|\|\| CGGGTTGGGTTAAAGGAAGCTTCTAATCAGAATGTCTACATTTTAAGATTAGATCTCAAA | 541 |
| Query | 541 | TCAGGTAGGACTACCCGCTGAACTTAAGCATATCAATAAGCGGAGGAAAAGAAACTAACA | 600 |
| Sbjct | 542 | \|\|\|\|\|\|\|\|\|\|\|\|\|\|\|\|\|\|\|\|\|\|\|\|\|\|\|\|\|\|\|\|\|\|\|\|\|\|\|\|\|\|\|\|\|\|\|\|\|\|\|\|\|\|\|\|\|\|\|\| TCAGGTAGGACTACCCGCTGAACTTAAGCATATCAATAAGCGGAGGAAAAGAAACTAACA | 601 |
| Query | 601 | AGGATTCCCCTAGTAGCGGCGAGCGAAGCGGGAAGAGCTCAAATTTATAATCTGGCACCT | 660 |
| Sbjct | 602 | \|\|\|\|\|\|\|\|\|\|\|\|\|\|\|\|\|\|\|\|\|\|\|\|\|\|\|\|\|\|\|\|\|\|\|\|\|\|\|\|\|\|\|\|\|\|\|\|\|\|\|\|\|\|\|\|\|\|\|\| AGGATTCCCCTAGTAGCGGCGAGCGAAGCGGGAAGAGCTCAAATTTATAATCTGGCACCT | 661 |
| Query | 661 | TCGGTGTCCGAGTTGTAATCTCTAGAAATGTTTTCCGCGTTGGACCGCACACAAGTCTGT | 720 |
| Sbjct | 662 | \|\|\|\|\|\|\|\|\|\|\|\|\|\|\|\|\|\|\|\|\|\|\|\|\|\|\|\|\|\|\|\|\|\|\|\|\|\|\|\|\|\|\|\|\|\|\|\|\|\|\|\|\|\|\|\|\|\|\|\| TCGGTGTCCGAGTTGTAATCTCTAGAAATGTTTTCCGCGTTGGACCGCACACAAGTCTGT | 721 |
| Query  Sbjct | 721  722 | TGGAATACAGCGGCATAGTGGTGAGACCCCCGTATATGGTGCGGACGCCCAGCG 774  \|\|\|\|\|\|\|\|\|\|\|\|\|\|\|\|\|\|\|\|\|\|\|\|\|\|\|\|\|\|\|\|\|\|\|\|\|\|\|\|\|\|\|\|\|\|\|\|\|\|\|\|\|\| TGGAATACAGCGGCATAGTGGTGAGACCCCCGTATATGGTGCGGACGCCCAGCG 775 |  |

**> KF411543 Rhodotorula mucilaginosa**

Length=1128

Score = 1397 bits (1548), Expect = 0.0 Identities = 774/774 (100%), Gaps = 0/774 (0%)

Strand=Plus/Plus

| Query | 1 | AGTGAATATAGGACGTCCAACTTAACTTGGAGTCCGAACTCTCACTTTCTAACCCTGTGC | 60 |
| --- | --- | --- | --- |
| Sbjct | 2 | \|\|\|\|\|\|\|\|\|\|\|\|\|\|\|\|\|\|\|\|\|\|\|\|\|\|\|\|\|\|\|\|\|\|\|\|\|\|\|\|\|\|\|\|\|\|\|\|\|\|\|\|\|\|\|\|\|\|\|\| AGTGAATATAGGACGTCCAACTTAACTTGGAGTCCGAACTCTCACTTTCTAACCCTGTGC | 61 |
| Query | 61 | ACTTGTTTGGGATAGTAACTCTCGCAAGAGAGCGAACTCCTATTCACTTATAAACACAAA | 120 |
| Sbjct | 62 | \|\|\|\|\|\|\|\|\|\|\|\|\|\|\|\|\|\|\|\|\|\|\|\|\|\|\|\|\|\|\|\|\|\|\|\|\|\|\|\|\|\|\|\|\|\|\|\|\|\|\|\|\|\|\|\|\|\|\|\| ACTTGTTTGGGATAGTAACTCTCGCAAGAGAGCGAACTCCTATTCACTTATAAACACAAA | 121 |
| Query | 121 | GTCTATGAATGTATTAAATTTTATAACAAAATAAAACTTTCAACAACGGATCTCTTGGCT | 180 |
| Sbjct | 122 | \|\|\|\|\|\|\|\|\|\|\|\|\|\|\|\|\|\|\|\|\|\|\|\|\|\|\|\|\|\|\|\|\|\|\|\|\|\|\|\|\|\|\|\|\|\|\|\|\|\|\|\|\|\|\|\|\|\|\|\| GTCTATGAATGTATTAAATTTTATAACAAAATAAAACTTTCAACAACGGATCTCTTGGCT | 181 |
| Query | 181 | CTCGCATCGATGAAGAACGCAGCGAAATGCGATAAGTAATGTGAATTGCAGAATTCAGTG | 240 |
| Sbjct | 182 | \|\|\|\|\|\|\|\|\|\|\|\|\|\|\|\|\|\|\|\|\|\|\|\|\|\|\|\|\|\|\|\|\|\|\|\|\|\|\|\|\|\|\|\|\|\|\|\|\|\|\|\|\|\|\|\|\|\|\|\| CTCGCATCGATGAAGAACGCAGCGAAATGCGATAAGTAATGTGAATTGCAGAATTCAGTG | 241 |
| Query | 241 | AATCATCGAATCTTTGAACGCACCTTGCGCTCCATGGTATTCCGTGGAGCATGCCTGTTT | 300 |
| Sbjct | 242 | \|\|\|\|\|\|\|\|\|\|\|\|\|\|\|\|\|\|\|\|\|\|\|\|\|\|\|\|\|\|\|\|\|\|\|\|\|\|\|\|\|\|\|\|\|\|\|\|\|\|\|\|\|\|\|\|\|\|\|\| AATCATCGAATCTTTGAACGCACCTTGCGCTCCATGGTATTCCGTGGAGCATGCCTGTTT | 301 |
| Query | 301 | GAGTGTCATGAATACTTCAACCCTCCTCTTTCTTAATGATTGAAGAGGTGTTTGGTTTCT | 360 |
| Sbjct | 302 | \|\|\|\|\|\|\|\|\|\|\|\|\|\|\|\|\|\|\|\|\|\|\|\|\|\|\|\|\|\|\|\|\|\|\|\|\|\|\|\|\|\|\|\|\|\|\|\|\|\|\|\|\|\|\|\|\|\|\|\| GAGTGTCATGAATACTTCAACCCTCCTCTTTCTTAATGATTGAAGAGGTGTTTGGTTTCT | 361 |
| Query | 361 | GAGCGCTGCTGGCCTTTACGGTCTAGCTCGTTCGTAATGCATTAGCATCCGCAATCGAAC | 420 |
| Sbjct | 362 | \|\|\|\|\|\|\|\|\|\|\|\|\|\|\|\|\|\|\|\|\|\|\|\|\|\|\|\|\|\|\|\|\|\|\|\|\|\|\|\|\|\|\|\|\|\|\|\|\|\|\|\|\|\|\|\|\|\|\|\|  GAGCGCTGCTGGCCTTTACGGTCTAGCTCGTTCGTAATGCATTAGCATCCGCAATCGAAC | 421 |

https://unite.ut.ee/cgi-bin/seq_search3.cgi 11/14

| 2017-6-7  Query | 421 | Blast output  TTCGGATTGACTTGGCGTAATAGACTATTCGCTGAGGAATTCTAGTCTTCGGATTAGAGC | 480 |
| --- | --- | --- | --- |
| Sbjct | 422 | \|\|\|\|\|\|\|\|\|\|\|\|\|\|\|\|\|\|\|\|\|\|\|\|\|\|\|\|\|\|\|\|\|\|\|\|\|\|\|\|\|\|\|\|\|\|\|\|\|\|\|\|\|\|\|\|\|\|\|\| TTCGGATTGACTTGGCGTAATAGACTATTCGCTGAGGAATTCTAGTCTTCGGATTAGAGC | 481 |
| Query | 481 | CGGGTTGGGTTAAAGGAAGCTTCTAATCAGAATGTCTACATTTTAAGATTAGATCTCAAA | 540 |
| Sbjct | 482 | \|\|\|\|\|\|\|\|\|\|\|\|\|\|\|\|\|\|\|\|\|\|\|\|\|\|\|\|\|\|\|\|\|\|\|\|\|\|\|\|\|\|\|\|\|\|\|\|\|\|\|\|\|\|\|\|\|\|\|\| CGGGTTGGGTTAAAGGAAGCTTCTAATCAGAATGTCTACATTTTAAGATTAGATCTCAAA | 541 |
| Query | 541 | TCAGGTAGGACTACCCGCTGAACTTAAGCATATCAATAAGCGGAGGAAAAGAAACTAACA | 600 |
| Sbjct | 542 | \|\|\|\|\|\|\|\|\|\|\|\|\|\|\|\|\|\|\|\|\|\|\|\|\|\|\|\|\|\|\|\|\|\|\|\|\|\|\|\|\|\|\|\|\|\|\|\|\|\|\|\|\|\|\|\|\|\|\|\| TCAGGTAGGACTACCCGCTGAACTTAAGCATATCAATAAGCGGAGGAAAAGAAACTAACA | 601 |
| Query | 601 | AGGATTCCCCTAGTAGCGGCGAGCGAAGCGGGAAGAGCTCAAATTTATAATCTGGCACCT | 660 |
| Sbjct | 602 | \|\|\|\|\|\|\|\|\|\|\|\|\|\|\|\|\|\|\|\|\|\|\|\|\|\|\|\|\|\|\|\|\|\|\|\|\|\|\|\|\|\|\|\|\|\|\|\|\|\|\|\|\|\|\|\|\|\|\|\| AGGATTCCCCTAGTAGCGGCGAGCGAAGCGGGAAGAGCTCAAATTTATAATCTGGCACCT | 661 |
| Query | 661 | TCGGTGTCCGAGTTGTAATCTCTAGAAATGTTTTCCGCGTTGGACCGCACACAAGTCTGT | 720 |
| Sbjct | 662 | \|\|\|\|\|\|\|\|\|\|\|\|\|\|\|\|\|\|\|\|\|\|\|\|\|\|\|\|\|\|\|\|\|\|\|\|\|\|\|\|\|\|\|\|\|\|\|\|\|\|\|\|\|\|\|\|\|\|\|\| TCGGTGTCCGAGTTGTAATCTCTAGAAATGTTTTCCGCGTTGGACCGCACACAAGTCTGT | 721 |
| Query  Sbjct | 721  722 | TGGAATACAGCGGCATAGTGGTGAGACCCCCGTATATGGTGCGGACGCCCAGCG 774  \|\|\|\|\|\|\|\|\|\|\|\|\|\|\|\|\|\|\|\|\|\|\|\|\|\|\|\|\|\|\|\|\|\|\|\|\|\|\|\|\|\|\|\|\|\|\|\|\|\|\|\|\|\| TGGAATACAGCGGCATAGTGGTGAGACCCCCGTATATGGTGCGGACGCCCAGCG 775 |  |

**> KF411537 Rhodotorula mucilaginosa**

Length=1147

Score = 1397 bits (1548), Expect = 0.0 Identities = 774/774 (100%), Gaps = 0/774 (0%)

Strand=Plus/Plus

| Query | 1 | AGTGAATATAGGACGTCCAACTTAACTTGGAGTCCGAACTCTCACTTTCTAACCCTGTGC | 60 |
| --- | --- | --- | --- |
| Sbjct | 11 | \|\|\|\|\|\|\|\|\|\|\|\|\|\|\|\|\|\|\|\|\|\|\|\|\|\|\|\|\|\|\|\|\|\|\|\|\|\|\|\|\|\|\|\|\|\|\|\|\|\|\|\|\|\|\|\|\|\|\|\| AGTGAATATAGGACGTCCAACTTAACTTGGAGTCCGAACTCTCACTTTCTAACCCTGTGC | 70 |
| Query | 61 | ACTTGTTTGGGATAGTAACTCTCGCAAGAGAGCGAACTCCTATTCACTTATAAACACAAA | 120 |
| Sbjct | 71 | \|\|\|\|\|\|\|\|\|\|\|\|\|\|\|\|\|\|\|\|\|\|\|\|\|\|\|\|\|\|\|\|\|\|\|\|\|\|\|\|\|\|\|\|\|\|\|\|\|\|\|\|\|\|\|\|\|\|\|\| ACTTGTTTGGGATAGTAACTCTCGCAAGAGAGCGAACTCCTATTCACTTATAAACACAAA | 130 |
| Query | 121 | GTCTATGAATGTATTAAATTTTATAACAAAATAAAACTTTCAACAACGGATCTCTTGGCT | 180 |
| Sbjct | 131 | \|\|\|\|\|\|\|\|\|\|\|\|\|\|\|\|\|\|\|\|\|\|\|\|\|\|\|\|\|\|\|\|\|\|\|\|\|\|\|\|\|\|\|\|\|\|\|\|\|\|\|\|\|\|\|\|\|\|\|\| GTCTATGAATGTATTAAATTTTATAACAAAATAAAACTTTCAACAACGGATCTCTTGGCT | 190 |
| Query | 181 | CTCGCATCGATGAAGAACGCAGCGAAATGCGATAAGTAATGTGAATTGCAGAATTCAGTG | 240 |
| Sbjct | 191 | \|\|\|\|\|\|\|\|\|\|\|\|\|\|\|\|\|\|\|\|\|\|\|\|\|\|\|\|\|\|\|\|\|\|\|\|\|\|\|\|\|\|\|\|\|\|\|\|\|\|\|\|\|\|\|\|\|\|\|\| CTCGCATCGATGAAGAACGCAGCGAAATGCGATAAGTAATGTGAATTGCAGAATTCAGTG | 250 |
| Query | 241 | AATCATCGAATCTTTGAACGCACCTTGCGCTCCATGGTATTCCGTGGAGCATGCCTGTTT | 300 |
| Sbjct | 251 | \|\|\|\|\|\|\|\|\|\|\|\|\|\|\|\|\|\|\|\|\|\|\|\|\|\|\|\|\|\|\|\|\|\|\|\|\|\|\|\|\|\|\|\|\|\|\|\|\|\|\|\|\|\|\|\|\|\|\|\| AATCATCGAATCTTTGAACGCACCTTGCGCTCCATGGTATTCCGTGGAGCATGCCTGTTT | 310 |
| Query | 301 | GAGTGTCATGAATACTTCAACCCTCCTCTTTCTTAATGATTGAAGAGGTGTTTGGTTTCT | 360 |
| Sbjct | 311 | \|\|\|\|\|\|\|\|\|\|\|\|\|\|\|\|\|\|\|\|\|\|\|\|\|\|\|\|\|\|\|\|\|\|\|\|\|\|\|\|\|\|\|\|\|\|\|\|\|\|\|\|\|\|\|\|\|\|\|\| GAGTGTCATGAATACTTCAACCCTCCTCTTTCTTAATGATTGAAGAGGTGTTTGGTTTCT | 370 |
| Query | 361 | GAGCGCTGCTGGCCTTTACGGTCTAGCTCGTTCGTAATGCATTAGCATCCGCAATCGAAC | 420 |
| Sbjct | 371 | \|\|\|\|\|\|\|\|\|\|\|\|\|\|\|\|\|\|\|\|\|\|\|\|\|\|\|\|\|\|\|\|\|\|\|\|\|\|\|\|\|\|\|\|\|\|\|\|\|\|\|\|\|\|\|\|\|\|\|\| GAGCGCTGCTGGCCTTTACGGTCTAGCTCGTTCGTAATGCATTAGCATCCGCAATCGAAC | 430 |
| Query | 421 | TTCGGATTGACTTGGCGTAATAGACTATTCGCTGAGGAATTCTAGTCTTCGGATTAGAGC | 480 |
| Sbjct | 431 | \|\|\|\|\|\|\|\|\|\|\|\|\|\|\|\|\|\|\|\|\|\|\|\|\|\|\|\|\|\|\|\|\|\|\|\|\|\|\|\|\|\|\|\|\|\|\|\|\|\|\|\|\|\|\|\|\|\|\|\| TTCGGATTGACTTGGCGTAATAGACTATTCGCTGAGGAATTCTAGTCTTCGGATTAGAGC | 490 |
| Query | 481 | CGGGTTGGGTTAAAGGAAGCTTCTAATCAGAATGTCTACATTTTAAGATTAGATCTCAAA | 540 |
| Sbjct | 491 | \|\|\|\|\|\|\|\|\|\|\|\|\|\|\|\|\|\|\|\|\|\|\|\|\|\|\|\|\|\|\|\|\|\|\|\|\|\|\|\|\|\|\|\|\|\|\|\|\|\|\|\|\|\|\|\|\|\|\|\| CGGGTTGGGTTAAAGGAAGCTTCTAATCAGAATGTCTACATTTTAAGATTAGATCTCAAA | 550 |
| Query | 541 | TCAGGTAGGACTACCCGCTGAACTTAAGCATATCAATAAGCGGAGGAAAAGAAACTAACA | 600 |
| Sbjct | 551 | \|\|\|\|\|\|\|\|\|\|\|\|\|\|\|\|\|\|\|\|\|\|\|\|\|\|\|\|\|\|\|\|\|\|\|\|\|\|\|\|\|\|\|\|\|\|\|\|\|\|\|\|\|\|\|\|\|\|\|\| TCAGGTAGGACTACCCGCTGAACTTAAGCATATCAATAAGCGGAGGAAAAGAAACTAACA | 610 |
| Query | 601 | AGGATTCCCCTAGTAGCGGCGAGCGAAGCGGGAAGAGCTCAAATTTATAATCTGGCACCT | 660 |
| Sbjct | 611 | \|\|\|\|\|\|\|\|\|\|\|\|\|\|\|\|\|\|\|\|\|\|\|\|\|\|\|\|\|\|\|\|\|\|\|\|\|\|\|\|\|\|\|\|\|\|\|\|\|\|\|\|\|\|\|\|\|\|\|\|  AGGATTCCCCTAGTAGCGGCGAGCGAAGCGGGAAGAGCTCAAATTTATAATCTGGCACCT | 670 |

https://unite.ut.ee/cgi-bin/seq_search3.cgi 12/14

| 2017-6-7  Query | Blast output  661 TCGGTGTCCGAGTTGTAATCTCTAGAAATGTTTTCCGCGTTGGACCGCACACAAGTCTGT | 720 |
| --- | --- | --- |
| Sbjct | \|\|\|\|\|\|\|\|\|\|\|\|\|\|\|\|\|\|\|\|\|\|\|\|\|\|\|\|\|\|\|\|\|\|\|\|\|\|\|\|\|\|\|\|\|\|\|\|\|\|\|\|\|\|\|\|\|\|\|\| 671 TCGGTGTCCGAGTTGTAATCTCTAGAAATGTTTTCCGCGTTGGACCGCACACAAGTCTGT | 730 |
| Query  Sbjct | 721 TGGAATACAGCGGCATAGTGGTGAGACCCCCGTATATGGTGCGGACGCCCAGCG 774  \|\|\|\|\|\|\|\|\|\|\|\|\|\|\|\|\|\|\|\|\|\|\|\|\|\|\|\|\|\|\|\|\|\|\|\|\|\|\|\|\|\|\|\|\|\|\|\|\|\|\|\|\|\|  731 TGGAATACAGCGGCATAGTGGTGAGACCCCCGTATATGGTGCGGACGCCCAGCG 784 |  |

**> KF411536 Rhodotorula mucilaginosa**

Length=1134

Score = 1397 bits (1548), Expect = 0.0 Identities = 774/774 (100%), Gaps = 0/774 (0%)

Strand=Plus/Plus

| Query | 1 | AGTGAATATAGGACGTCCAACTTAACTTGGAGTCCGAACTCTCACTTTCTAACCCTGTGC | 60 |
| --- | --- | --- | --- |
| Sbjct | 5 | \|\|\|\|\|\|\|\|\|\|\|\|\|\|\|\|\|\|\|\|\|\|\|\|\|\|\|\|\|\|\|\|\|\|\|\|\|\|\|\|\|\|\|\|\|\|\|\|\|\|\|\|\|\|\|\|\|\|\|\| AGTGAATATAGGACGTCCAACTTAACTTGGAGTCCGAACTCTCACTTTCTAACCCTGTGC | 64 |
| Query | 61 | ACTTGTTTGGGATAGTAACTCTCGCAAGAGAGCGAACTCCTATTCACTTATAAACACAAA | 120 |
| Sbjct | 65 | \|\|\|\|\|\|\|\|\|\|\|\|\|\|\|\|\|\|\|\|\|\|\|\|\|\|\|\|\|\|\|\|\|\|\|\|\|\|\|\|\|\|\|\|\|\|\|\|\|\|\|\|\|\|\|\|\|\|\|\| ACTTGTTTGGGATAGTAACTCTCGCAAGAGAGCGAACTCCTATTCACTTATAAACACAAA | 124 |
| Query | 121 | GTCTATGAATGTATTAAATTTTATAACAAAATAAAACTTTCAACAACGGATCTCTTGGCT | 180 |
| Sbjct | 125 | \|\|\|\|\|\|\|\|\|\|\|\|\|\|\|\|\|\|\|\|\|\|\|\|\|\|\|\|\|\|\|\|\|\|\|\|\|\|\|\|\|\|\|\|\|\|\|\|\|\|\|\|\|\|\|\|\|\|\|\| GTCTATGAATGTATTAAATTTTATAACAAAATAAAACTTTCAACAACGGATCTCTTGGCT | 184 |
| Query | 181 | CTCGCATCGATGAAGAACGCAGCGAAATGCGATAAGTAATGTGAATTGCAGAATTCAGTG | 240 |
| Sbjct | 185 | \|\|\|\|\|\|\|\|\|\|\|\|\|\|\|\|\|\|\|\|\|\|\|\|\|\|\|\|\|\|\|\|\|\|\|\|\|\|\|\|\|\|\|\|\|\|\|\|\|\|\|\|\|\|\|\|\|\|\|\| CTCGCATCGATGAAGAACGCAGCGAAATGCGATAAGTAATGTGAATTGCAGAATTCAGTG | 244 |
| Query | 241 | AATCATCGAATCTTTGAACGCACCTTGCGCTCCATGGTATTCCGTGGAGCATGCCTGTTT | 300 |
| Sbjct | 245 | \|\|\|\|\|\|\|\|\|\|\|\|\|\|\|\|\|\|\|\|\|\|\|\|\|\|\|\|\|\|\|\|\|\|\|\|\|\|\|\|\|\|\|\|\|\|\|\|\|\|\|\|\|\|\|\|\|\|\|\| AATCATCGAATCTTTGAACGCACCTTGCGCTCCATGGTATTCCGTGGAGCATGCCTGTTT | 304 |
| Query | 301 | GAGTGTCATGAATACTTCAACCCTCCTCTTTCTTAATGATTGAAGAGGTGTTTGGTTTCT | 360 |
| Sbjct | 305 | \|\|\|\|\|\|\|\|\|\|\|\|\|\|\|\|\|\|\|\|\|\|\|\|\|\|\|\|\|\|\|\|\|\|\|\|\|\|\|\|\|\|\|\|\|\|\|\|\|\|\|\|\|\|\|\|\|\|\|\| GAGTGTCATGAATACTTCAACCCTCCTCTTTCTTAATGATTGAAGAGGTGTTTGGTTTCT | 364 |
| Query | 361 | GAGCGCTGCTGGCCTTTACGGTCTAGCTCGTTCGTAATGCATTAGCATCCGCAATCGAAC | 420 |
| Sbjct | 365 | \|\|\|\|\|\|\|\|\|\|\|\|\|\|\|\|\|\|\|\|\|\|\|\|\|\|\|\|\|\|\|\|\|\|\|\|\|\|\|\|\|\|\|\|\|\|\|\|\|\|\|\|\|\|\|\|\|\|\|\| GAGCGCTGCTGGCCTTTACGGTCTAGCTCGTTCGTAATGCATTAGCATCCGCAATCGAAC | 424 |
| Query | 421 | TTCGGATTGACTTGGCGTAATAGACTATTCGCTGAGGAATTCTAGTCTTCGGATTAGAGC | 480 |
| Sbjct | 425 | \|\|\|\|\|\|\|\|\|\|\|\|\|\|\|\|\|\|\|\|\|\|\|\|\|\|\|\|\|\|\|\|\|\|\|\|\|\|\|\|\|\|\|\|\|\|\|\|\|\|\|\|\|\|\|\|\|\|\|\| TTCGGATTGACTTGGCGTAATAGACTATTCGCTGAGGAATTCTAGTCTTCGGATTAGAGC | 484 |
| Query | 481 | CGGGTTGGGTTAAAGGAAGCTTCTAATCAGAATGTCTACATTTTAAGATTAGATCTCAAA | 540 |
| Sbjct | 485 | \|\|\|\|\|\|\|\|\|\|\|\|\|\|\|\|\|\|\|\|\|\|\|\|\|\|\|\|\|\|\|\|\|\|\|\|\|\|\|\|\|\|\|\|\|\|\|\|\|\|\|\|\|\|\|\|\|\|\|\| CGGGTTGGGTTAAAGGAAGCTTCTAATCAGAATGTCTACATTTTAAGATTAGATCTCAAA | 544 |
| Query | 541 | TCAGGTAGGACTACCCGCTGAACTTAAGCATATCAATAAGCGGAGGAAAAGAAACTAACA | 600 |
| Sbjct | 545 | \|\|\|\|\|\|\|\|\|\|\|\|\|\|\|\|\|\|\|\|\|\|\|\|\|\|\|\|\|\|\|\|\|\|\|\|\|\|\|\|\|\|\|\|\|\|\|\|\|\|\|\|\|\|\|\|\|\|\|\| TCAGGTAGGACTACCCGCTGAACTTAAGCATATCAATAAGCGGAGGAAAAGAAACTAACA | 604 |
| Query | 601 | AGGATTCCCCTAGTAGCGGCGAGCGAAGCGGGAAGAGCTCAAATTTATAATCTGGCACCT | 660 |
| Sbjct | 605 | \|\|\|\|\|\|\|\|\|\|\|\|\|\|\|\|\|\|\|\|\|\|\|\|\|\|\|\|\|\|\|\|\|\|\|\|\|\|\|\|\|\|\|\|\|\|\|\|\|\|\|\|\|\|\|\|\|\|\|\| AGGATTCCCCTAGTAGCGGCGAGCGAAGCGGGAAGAGCTCAAATTTATAATCTGGCACCT | 664 |
| Query | 661 | TCGGTGTCCGAGTTGTAATCTCTAGAAATGTTTTCCGCGTTGGACCGCACACAAGTCTGT | 720 |
| Sbjct | 665 | \|\|\|\|\|\|\|\|\|\|\|\|\|\|\|\|\|\|\|\|\|\|\|\|\|\|\|\|\|\|\|\|\|\|\|\|\|\|\|\|\|\|\|\|\|\|\|\|\|\|\|\|\|\|\|\|\|\|\|\| TCGGTGTCCGAGTTGTAATCTCTAGAAATGTTTTCCGCGTTGGACCGCACACAAGTCTGT | 724 |
| Query  Sbjct | 721  725 | TGGAATACAGCGGCATAGTGGTGAGACCCCCGTATATGGTGCGGACGCCCAGCG 774  \|\|\|\|\|\|\|\|\|\|\|\|\|\|\|\|\|\|\|\|\|\|\|\|\|\|\|\|\|\|\|\|\|\|\|\|\|\|\|\|\|\|\|\|\|\|\|\|\|\|\|\|\|\| TGGAATACAGCGGCATAGTGGTGAGACCCCCGTATATGGTGCGGACGCCCAGCG 778 |  |

| Lambda | K | H |
| --- | --- | --- |
| 0.634 | 0.408 | 0.912 |
| Gapped Lambda | K | H |

https://unite.ut.ee/cgi-bin/seq_search3.cgi 13/14

2017-6-7 Blast output

https://unite.ut.ee/cgi-bin/seq_search3.cgi 14/14
