# Supplementary material for: Whole genome sequencing of Rhodotorula mucilaginosa isolated from the chewing stick (Distemonanthus benthamianus): insights into Rhodotorula phylogeny, mitogenome dynamics and carotenoid biosynthesis
Source: PeerJ. 2017 Nov 14;5:e4030. doi: 10.7717/peerj.4030 (PMC5691792; doi:10.7717/peerj.4030)
Supplement: Table S2 [file peerj-05-4030-s002.doc]

Supplemental Table 2: Identification of proteins associated with the biosynthesis of carotenoid in *Rhodotorula* and *Spriobolus*

| Gene ID | Organism | Contig_ID | Enzyme | HMM profile | E-value |
| --- | --- | --- | --- | --- | --- |
| AEVR02~1972_g | Rhodotorula toruloides ATCC204091 | AEVR02000018 | phytoene desaturase | TIGR02734 | 5.30E-128 |
| AEVR02~1975_g | Rhodotorula toruloides ATCC204091 | AEVR02000018 | Squalene/Phytoene synthase | PF00494.18 | 9.00E-040 |
| AEVR02~1975_g | Rhodotorula toruloides ATCC204091 | AEVR02000018 | lycopene cyclase | TIGR03462 | 7.50E-035 |
| AEVR02~2232_g | Rhodotorula toruloides ATCC204091 | AEVR02000018 | Isopentenyl-diphosphate delta-isomerase | TIGR02150 | 1.40E-047 |
| AEVR02~241_g | Rhodotorula toruloides ATCC204091 | AEVR02000013 | Squalene/Phytoene synthase | PF00494.18 | 3.30E-039 |
| AEVR02~3144_g | Rhodotorula toruloides ATCC204091 | AEVR02000002 | Geranyl pyrophosphate synthase (IspA) | PF00348.16 | 3.20E-096 |
| AEVR02~5172_g | Rhodotorula toruloides ATCC204091 | AEVR02000017 | Squalene/Phytoene synthase | PF00494.18 | 2.60E-042 |
| AJMJ01~194_g | Rhodotorula toruloides MTCC457 | JH650665 | Geranyl pyrophosphate synthase (IspA) | PF00348.16 | 6.80E-096 |
| AJMJ01~3838_g | Rhodotorula toruloides MTCC457 | JH650732 | phytoene desaturase | TIGR02734 | 8.30E-130 |
| AJMJ01~3841_g | Rhodotorula toruloides MTCC457 | JH650732 | Squalene/Phytoene synthase | PF00494.18 | 4.80E-040 |
| AJMJ01~3841_g | Rhodotorula toruloides MTCC457 | JH650732 | lycopene cyclase | TIGR03462 | 1.80E-035 |
| AJMJ01~7700_g | Rhodotorula toruloides MTCC457 | JH650719 | Isopentenyl-diphosphate delta-isomerase | TIGR02150 | 1.40E-047 |
| AJMJ01~7732_g | Rhodotorula toruloides MTCC457 | JH650719 | Squalene/Phytoene synthase | PF00494.18 | 2.90E-044 |
| AJMJ01~802_g | Rhodotorula toruloides MTCC457 | AJMJ01000482 | Squalene/Phytoene synthase | PF00494.18 | 3.20E-038 |
| ALAU01~1654_g | Rhodotorula toruloides NP11 | ALAU01000150 | Geranyl pyrophosphate synthase (IspA) | PF00348.16 | 6.80E-096 |
| ALAU01~1772_g | Rhodotorula toruloides NP11 | ALAU01000186 | Squalene/Phytoene synthase | PF00494.18 | 2.90E-044 |
| ALAU01~5147_g | Rhodotorula toruloides NP11 | ALAU01000174 | Squalene/Phytoene synthase | PF00494.18 | 3.20E-038 |
| ALAU01~5662_g | Rhodotorula toruloides NP11 | ALAU01000209 | Isopentenyl-diphosphate delta-isomerase | TIGR02150 | 1.80E-047 |
| ALAU01~7694_g | Rhodotorula toruloides NP11 | ALAU01000037 | phytoene desaturase | TIGR02734 | 8.30E-130 |
| ALAU01~7697_g | Rhodotorula toruloides NP11 | ALAU01000037 | Squalene/Phytoene synthase | PF00494.18 | 4.80E-040 |
| ALAU01~7697_g | Rhodotorula toruloides NP11 | ALAU01000037 | lycopene cyclase | TIGR03462 | 1.80E-035 |
| BCIY01~2014_g | Rhodotorula toruloides JCM10020 | BCIY01000019 | Squalene/Phytoene synthase | PF00494.18 | 2.90E-044 |
| BCIY01~3431_g | Rhodotorula toruloides JCM10020 | BCIY01000003 | phytoene desaturase | TIGR02734 | 8.30E-130 |
| BCIY01~3434_g | Rhodotorula toruloides JCM10020 | BCIY01000003 | Squalene/Phytoene synthase | PF00494.18 | 4.80E-040 |
| BCIY01~3434_g | Rhodotorula toruloides JCM10020 | BCIY01000003 | lycopene cyclase | TIGR03462 | 1.80E-035 |
| BCIY01~5908_g | Rhodotorula toruloides JCM10020 | BCIY01000002 | Isopentenyl-diphosphate delta-isomerase | TIGR02150 | 1.80E-047 |
| BCIY01~5910_g | Rhodotorula toruloides JCM10020 | BCIY01000002 | Isopentenyl-diphosphate delta-isomerase | TIGR02150 | 1.80E-047 |
| BCIY01~6299_g | Rhodotorula toruloides JCM10020 | BCIY01000002 | Squalene/Phytoene synthase | PF00494.18 | 3.20E-038 |
| BCIY01~876_g | Rhodotorula toruloides JCM10020 | BCIY01000015 | Geranyl pyrophosphate synthase (IspA) | PF00348.16 | 6.80E-096 |
| BCIZ01~154_g | Rhodotorula toruloides JCM10021 | BCIZ01000012 | Squalene/Phytoene synthase | PF00494.18 | 2.60E-042 |
| BCIZ01~336_g | Rhodotorula toruloides JCM10021 | BCIZ01000014 | Squalene/Phytoene synthase | PF00494.18 | 3.30E-039 |
| BCIZ01~3382_g | Rhodotorula toruloides JCM10021 | BCIZ01000011 | Isopentenyl-diphosphate delta-isomerase | TIGR02150 | 1.40E-047 |
| BCIZ01~6297_g | Rhodotorula toruloides JCM10021 | BCIZ01000006 | Squalene/Phytoene synthase | PF00494.18 | 9.00E-040 |
| BCIZ01~6297_g | Rhodotorula toruloides JCM10021 | BCIZ01000006 | lycopene cyclase | TIGR03462 | 7.50E-035 |
| BCIZ01~6300_g | Rhodotorula toruloides JCM10021 | BCIZ01000006 | phytoene desaturase | TIGR02734 | 5.30E-128 |
| BCIZ01~960_g | Rhodotorula toruloides JCM10021 | BCIZ01000002 | Geranyl pyrophosphate synthase (IspA) | PF00348.16 | 3.20E-096 |
| BCJA01~3320_g | Rhodotorula toruloides JCM10049 | BCJA01000017 | Squalene/Phytoene synthase | PF00494.18 | 4.80E-040 |
| BCJA01~3320_g | Rhodotorula toruloides JCM10049 | BCJA01000017 | lycopene cyclase | TIGR03462 | 1.80E-035 |
| BCJA01~3323_g | Rhodotorula toruloides JCM10049 | BCJA01000017 | phytoene desaturase | TIGR02734 | 2.40E-129 |
| BCJA01~379_g | Rhodotorula toruloides JCM10049 | BCJA01000018 | Isopentenyl-diphosphate delta-isomerase | TIGR02150 | 1.80E-047 |
| BCJA01~4535_g | Rhodotorula toruloides JCM10049 | BCJA01000013 | Geranyl pyrophosphate synthase (IspA) | PF00348.16 | 2.00E-095 |
| BCJA01~7178_g | Rhodotorula toruloides JCM10049 | BCJA01000009 | Squalene/Phytoene synthase | PF00494.18 | 3.20E-038 |
| BCJA01~7433_g | Rhodotorula toruloides JCM10049 | BCJA01000015 | Squalene/Phytoene synthase | PF00494.18 | 2.90E-044 |
| BCJE01~1158_g | Rhodotorula toruloides JCM10049 | BCJE01000020 | Squalene/Phytoene synthase | PF00494.18 | 1.60E-043 |
| BCJE01~2664_g | Rhodotorula toruloides JCM10049 | BCJE01000009 | Isopentenyl-diphosphate delta-isomerase | TIGR02150 | 1.20E-047 |
| BCJE01~2917_g | Rhodotorula toruloides JCM10049 | BCJE01000009 | Squalene/Phytoene synthase | PF00494.18 | 6.20E-040 |
| BCJE01~2917_g | Rhodotorula toruloides JCM10049 | BCJE01000009 | lycopene cyclase | TIGR03462 | 2.40E-034 |
| BCJE01~2920_g | Rhodotorula toruloides JCM10049 | BCJE01000009 | phytoene desaturase | TIGR02734 | 2.80E-128 |
| BCJE01~4236_g | Rhodotorula toruloides JCM10049 | BCJE01000012 | Geranyl pyrophosphate synthase (IspA) | PF00348.16 | 1.50E-095 |
| BCJE01~6808_g | Rhodotorula toruloides JCM10049 | BCJE01000008 | Squalene/Phytoene synthase | PF00494.18 | 1.10E-039 |
| CENE01~1370_g | Sporidiobolus salmonicolor CBS6832 | CENE01000012 | Squalene/Phytoene synthase | PF00494.18 | 2.30E-044 |
| CENE01~1370_g | Sporidiobolus salmonicolor CBS6832 | CENE01000012 | lycopene cyclase | TIGR03462 | 6.90E-035 |
| CENE01~1371_g | Sporidiobolus salmonicolor CBS6832 | CENE01000012 | phytoene desaturase | TIGR02734 | 5.40E-125 |
| CENE01~2336_g | Sporidiobolus salmonicolor CBS6832 | CENE01000010 | Squalene/Phytoene synthase | PF00494.18 | 3.50E-044 |
| CENE01~2482_g | Sporidiobolus salmonicolor CBS6832 | CENE01000016 | Geranyl pyrophosphate synthase (IspA) | PF00348.16 | 1.20E-094 |
| CENE01~4516_g | Sporidiobolus salmonicolor CBS6832 | CENE01000001 | Squalene/Phytoene synthase | PF00494.18 | 1.30E-036 |
| CENE01~6711_g | Sporidiobolus salmonicolor CBS6832 | CENE01000037 | Isopentenyl-diphosphate delta-isomerase | TIGR02150 | 5.40E-048 |
| CWKI01~3571_g | Rhodosporidium toruloides IFO0880 | CWKI01000008 | Isopentenyl-diphosphate delta-isomerase | TIGR02150 | 1.40E-047 |
| CWKI01~4059_g | Rhodosporidium toruloides IFO0880 | CWKI01000013 | Squalene/Phytoene synthase | PF00494.18 | 2.60E-042 |
| CWKI01~429_g | Rhodosporidium toruloides IFO0880 | CWKI01000003 | Geranyl pyrophosphate synthase (IspA) | PF00348.16 | 3.20E-096 |
| CWKI01~4399_g | Rhodosporidium toruloides IFO0880 | CWKI01000001 | phytoene desaturase | TIGR02734 | 5.30E-128 |
| CWKI01~4402_g | Rhodosporidium toruloides IFO0880 | CWKI01000001 | Squalene/Phytoene synthase | PF00494.18 | 9.00E-040 |
| CWKI01~4402_g | Rhodosporidium toruloides IFO0880 | CWKI01000001 | lycopene cyclase | TIGR03462 | 7.50E-035 |
| CWKI01~7315_g | Rhodosporidium toruloides IFO0880 | CWKI01000009 | Squalene/Phytoene synthase | PF00494.18 | 3.30E-039 |
| JTAO01~1854_g | Rhodotorula graminis WP1 | JTAO01000278 | Squalene/Phytoene synthase | PF00494.18 | 2.40E-043 |
| JTAO01~2422_g | Rhodotorula graminis WP1 | JTAO01000020 | Squalene/Phytoene synthase | PF00494.18 | 1.70E-033 |
| JTAO01~2538_g | Rhodotorula graminis WP1 | JTAO01000149 | Squalene/Phytoene synthase | PF00494.18 | 4.50E-045 |
| JTAO01~2538_g | Rhodotorula graminis WP1 | JTAO01000149 | lycopene cyclase | TIGR03462 | 2.80E-035 |
| JTAO01~2540_g | Rhodotorula graminis WP1 | JTAO01000149 | phytoene desaturase | TIGR02734 | 2.30E-124 |
| JTAO01~5118_g | Rhodotorula graminis WP1 | JTAO01000158 | Isopentenyl-diphosphate delta-isomerase | TIGR02150 | 8.60E-047 |
| JTAO01~901_g | Rhodotorula graminis WP1 | JTAO01000169 | Geranyl pyrophosphate synthase (IspA) | PF00348.16 | 2.00E-096 |
| JWTJ~2884_g | Rhodotorula mucilaginosa C25t1 | JWTJ01000382 | Squalene/Phytoene synthase | PF00494.18 | 2.30E-042 |
| JWTJ~2884_g | Rhodotorula mucilaginosa C25t1 | JWTJ01000382 | lycopene cyclase | TIGR03462 | 2.40E-034 |
| JWTJ~2887_g | Rhodotorula mucilaginosa C25t1 | JWTJ01000382 | phytoene desaturase | TIGR02734 | 2.00E-116 |
| JWTJ~3392_g | Rhodotorula mucilaginosa C25t1 | JWTJ01000703 | Isopentenyl-diphosphate delta-isomerase | TIGR02150 | 4.10E-048 |
| JWTJ~4579_g | Rhodotorula mucilaginosa C25t1 | JWTJ01000911 | Geranyl pyrophosphate synthase (IspA) | PF00348.16 | 3.00E-090 |
| JWTJ~6094_g | Rhodotorula mucilaginosa C25t1 | JWTJ01000355 | Squalene/Phytoene synthase | PF00494.18 | 2.00E-045 |
| JWTJ~851_g | Rhodotorula mucilaginosa C25t1 | JWTJ01000274 | Squalene/Phytoene synthase | PF00494.18 | 6.60E-039 |
| LCTU01~2895_g | Rhodotorula toruloides IFO0559 | LCTU01000114 | Squalene/Phytoene synthase | PF00494.18 | 2.90E-044 |
| LCTU01~4115_g | Rhodotorula toruloides IFO0559 | LCTU01000005 | Geranyl pyrophosphate synthase (IspA) | PF00348.16 | 6.80E-096 |
| LCTU01~5286_g | Rhodotorula toruloides IFO0559 | LCTU01000048 | phytoene desaturase | TIGR02734 | 8.30E-130 |
| LCTU01~5289_g | Rhodotorula toruloides IFO0559 | LCTU01000048 | Squalene/Phytoene synthase | PF00494.18 | 4.80E-040 |
| LCTU01~5289_g | Rhodotorula toruloides IFO0559 | LCTU01000048 | lycopene cyclase | TIGR03462 | 1.80E-035 |
| LCTU01~6639_g | Rhodotorula toruloides IFO0559 | LCTU01000033 | Squalene/Phytoene synthase | PF00494.18 | 3.20E-038 |
| LCTU01~7453_g | Rhodotorula toruloides IFO0559 | LCTU01000052 | Isopentenyl-diphosphate delta-isomerase | TIGR02150 | 1.80E-047 |
| LCTV01~1457_g | Rhodotorula toruloides IFO0880 | LCTV01000060 | Geranyl pyrophosphate synthase (IspA) | PF00348.16 | 3.20E-096 |
| LCTV01~3172_g | Rhodotorula toruloides IFO0880 | LCTV01000039 | Isopentenyl-diphosphate delta-isomerase | TIGR02150 | 1.40E-047 |
| LCTV01~4540_g | Rhodotorula toruloides IFO0880 | LCTV01000049 | phytoene desaturase | TIGR02734 | 5.30E-128 |
| LCTV01~4543_g | Rhodotorula toruloides IFO0880 | LCTV01000049 | Squalene/Phytoene synthase | PF00494.18 | 9.00E-040 |
| LCTV01~4543_g | Rhodotorula toruloides IFO0880 | LCTV01000049 | lycopene cyclase | TIGR03462 | 7.50E-035 |
| LCTV01~6573_g | Rhodotorula toruloides IFO0880 | LCTV01000052 | Squalene/Phytoene synthase | PF00494.18 | 3.30E-039 |
| LCTV01~7437_g | Rhodotorula toruloides IFO0880 | LCTV01000013 | Squalene/Phytoene synthase | PF00494.18 | 2.60E-042 |
| LNKU01~12_g | Rhodotorula toruloides ATCC10657 | LNKU01000018 | Squalene/Phytoene synthase | PF00494.18 | 2.60E-042 |
| LNKU01~2063_g | Rhodotorula toruloides ATCC10657 | LNKU01000020 | Isopentenyl-diphosphate delta-isomerase | TIGR02150 | 1.40E-047 |
| LNKU01~6064_g | Rhodotorula toruloides ATCC10657 | LNKU01000010 | phytoene desaturase | TIGR02734 | 5.30E-128 |
| LNKU01~6067_g | Rhodotorula toruloides ATCC10657 | LNKU01000010 | Squalene/Phytoene synthase | PF00494.18 | 9.00E-040 |
| LNKU01~6067_g | Rhodotorula toruloides ATCC10657 | LNKU01000010 | lycopene cyclase | TIGR03462 | 7.50E-035 |
| LNKU01~6619_g | Rhodotorula toruloides ATCC10657 | LNKU01000001 | Squalene/Phytoene synthase | PF00494.18 | 3.30E-039 |
| LNKU01~7567_g | Rhodotorula toruloides ATCC10657 | LNKU01000008 | Geranyl pyrophosphate synthase (IspA) | PF00348.16 | 3.20E-096 |
| LNQQ01~3286_g | Rhodotorula toruloides ATCC10788 | LNQQ01000010 | Geranyl pyrophosphate synthase (IspA) | PF00348.16 | 6.80E-096 |
| LNQQ01~5924_g | Rhodotorula toruloides ATCC10788 | LNQQ01000001 | Isopentenyl-diphosphate delta-isomerase | TIGR02150 | 1.80E-047 |
| LNQQ01~6309_g | Rhodotorula toruloides ATCC10788 | LNQQ01000001 | Squalene/Phytoene synthase | PF00494.18 | 3.20E-038 |
| LNQQ01~6976_g | Rhodotorula toruloides ATCC10788 | LNQQ01000004 | Squalene/Phytoene synthase | PF00494.18 | 4.80E-040 |
| LNQQ01~6976_g | Rhodotorula toruloides ATCC10788 | LNQQ01000004 | lycopene cyclase | TIGR03462 | 1.80E-035 |
| LNQQ01~6979_g | Rhodotorula toruloides ATCC10788 | LNQQ01000004 | phytoene desaturase | TIGR02734 | 8.30E-130 |
| LNQQ01~7604_g | Rhodotorula toruloides ATCC10788 | LNQQ01000012 | Squalene/Phytoene synthase | PF00494.18 | 2.90E-044 |
| LQXB01~1689_g | Rhodotorula sp JG 1b | LQXB01000011 | Squalene/Phytoene synthase | PF00494.18 | 4.50E-039 |
| LQXB01~2317_g | Rhodotorula sp JG 1b | LQXB01000016 | Squalene/Phytoene synthase | PF00494.18 | 7.10E-044 |
| LQXB01~2317_g | Rhodotorula sp JG 1b | LQXB01000016 | lycopene cyclase | TIGR03462 | 6.40E-032 |
| LQXB01~2320_g | Rhodotorula sp JG 1b | LQXB01000016 | phytoene desaturase | TIGR02734 | 9.90E-126 |
| LQXB01~3209_g | Rhodotorula sp JG 1b | LQXB01000027 | Isopentenyl-diphosphate delta-isomerase | TIGR02150 | 4.00E-048 |
| LQXB01~3984_g | Rhodotorula sp JG 1b | LQXB01000047 | Geranyl pyrophosphate synthase (IspA) | PF00348.16 | 5.20E-093 |
| LQXB01~864_g | Rhodotorula sp JG 1b | LQXB01000026 | Squalene/Phytoene synthase | PF00494.18 | 7.40E-046 |
| NIUW01~4583_g | Rhodotorula mucilaginosa RIT389 | NIUW01000001 | Squalene/Phytoene synthase | PF00494.18 | 7.10E-039 |
| NIUW01~5781_g | Rhodotorula mucilaginosa RIT389 | NIUW01000009 | Isopentenyl-diphosphate delta-isomerase | TIGR02150 | 3.90E-048 |
| NIUW01~713_g | Rhodotorula mucilaginosa RIT389 | NIUW01000053 | Squalene/Phytoene synthase | PF00494.18 | 4.20E-044 |
| NIUW01~713_g | Rhodotorula mucilaginosa RIT389 | NIUW01000053 | lycopene cyclase | TIGR03462 | 3.00E-032 |
